# A new two-parameter mixture family of generalized distributions: Statistical properties and application

**DOI:** 10.1016/j.heliyon.2024.e38198

**Published:** 2024-09-24

**Authors:** Alaa R. El-Alosey, Mohammed S. Alotaibi, Ahmed M. Gemeay

**Affiliations:** Department of Mathematics, Faculty of Science, Tanta University, Tanta 31527, Egypt

**Keywords:** G-family of distributions, Mixtures of distributions, Moments, Quantile function, Numerical simulation

## Abstract

This paper introduces a novel two-parameter G-family of distributions derived through a mixing approach, where a mixture of the exponentiated G-family with parameter *α* follows gamma distribution. Within this new family, we focus on the mixture of gamma-exponentiated exponential distribution (MGExED) as a key sub-model. The study thoroughly investigates by determining some of the MGExED statistical properties, such as quantile function, moments, and order statistics. The MGExED's parameters are estimated using different estimation methods. Due to the complexity of obtaining explicit forms for MGExED's estimators, the accuracy of these estimates is assessed through Monte Carlo simulations. To illustrate the practical utility of the MGExED, we apply it to two distinct real datasets, demonstrating its superior flexibility and fit compared to several established distributions in the literature. This comprehensive evaluation underscores the MGExED's potential for broader statistical modeling and analysis application.

## 1 Introduction

Using base and mixture probability distributions in financial modeling has long been a fundamental practice, offering profound insights into the intricate behaviors of financial markets. Numerous studies have delved into the application of diverse probability distributions within the domain of finance. A mixed distribution involves amalgamating two or more distinct probability distributions in statistics. It represents a statistical population comprising subpopulations, where the weights correspond to the proportions of each subpopulation within the overall population, and the mixture of probability density components represents the density functions of these subpopulations. These subpopulations can encompass univariate or multivariate distributions, discrete or continuous, and may originate from distinct or identical distribution families with varying parameters. Applying a mixed distribution becomes pertinent when a dataset exhibits discrete subgroups with distinct characteristics that warrant independent description.

In recent years, considerable attention has been directed toward the challenge of creating mixing distributions derived from many distributions. For instance, Roy et al. [30] explored the Poisson mixture of the binomial distribution, while Wood [36] introduced a cumulative distribution function-based approach to formulate the mixture of the binomial distribution. The domain of finance has witnessed the development of binomial mixtures involving various distributions, such as Poisson, normal, chi-squared, F, t, beta, gamma, exponential, rectangular, and Erlang, as investigated by Roy et al. [31]. Zhu et al. [37] recently employed a beta-binomial-Poisson mixture distribution to simultaneously model the number of successes and the number of binary trials. Shkedy et al. [32] devised the hierarchical binomial-Poisson model to analyze correlated binary data in a crossover design, considering the Poisson nature of the number of responses and the dose-dependent number of trials. Grilli et al. [15] utilized a binomial finite mixture model to predict the number of credits obtained by first-year students at the University of Florence's School of Economics. Knape et al. [20] assessed the sensitivity of binomial N-mixture models to over-dispersion in abundance and detection, employing simulations and a case study. El-Alosey [12] introduced the binomial-exponential mixture by deriving the probability mass function of discrete mixtures of distributions using the probability-generating function approach. Abed Al-Kadim and Al-Hussani [4] created the binomial mixture of the Erlang distribution using the moment's technique and Laplace transform. McLachlan et al. [23] demonstrated the effectiveness of mixture distributions in modeling the multimodal characteristics of financial returns. Furthermore, recent contributions have expanded the realm of mixing distributions. Adnan and Kiser [19] introduced Triple Binomials, defined as a multiplicative mixing of the identical three distributions. El-Alosey and Eledum [13] introduced binomial-discrete Erlang-truncated exponential mixtures, discussing their application in the context of cancer disease. These developments underscore the growing interest and significance of mixing distributions in financial modeling and related fields, offering diverse and flexible tools for capturing complex data patterns and underlying statistical relationships.

Numerous families of distributions have emerged through the extension of well-established distributions by incorporating one or more parameters into their foundational structures. These generalized distributions offer flexibility in modeling various data types, catering to diverse economic, engineering, lifetime analysis, insurance, finance, and environmental sciences applications. Many extensively explored distribution families have exhibited augmented adaptability, contributing significantly to statistical modeling techniques. Noteworthy among these advancements are the exponentiated-G (E-G) class introduced by Mudholkar et al. [24], the beta-G class proposed by Eugene et al. [14], the gamma-G distributions formulated by Zografos and Balakrishnan [38], and the Kumaraswamy Weibull-G distribution introduced by Cordeiro et al. in [11]. Additionally, Cordeiro and Castro [10] introduced the Kumaraswamy-G distribution, Alzaatreh et al. [5] presented the transform-transformer distribution and Bello et al. [7] established the type II Half-Logistic exponentiated-G distribution. Notably, Kajuru et al. [16] contributed to this expansion by introducing the odd Gompertz-Exponential distribution.

Extending existing distributions to form diverse families has proven invaluable in statistical modeling, catering to a broad spectrum of real-world applications. By introducing additional parameters to well-known distributions, researchers have unlocked a plethora of new distributions, facilitating enhanced adaptability and refined modeling capabilities across various disciplines. The evolution and investigation of these extended distribution families have garnered substantial attention in the scientific community due to their versatility and applicability in diverse fields. The exhaustive examination and utilization of such distributions underscore their significance in improving the adaptability and efficacy of statistical models, serving as vital tools in empirical studies and practical applications.

The remainder of this paper is structured as follows: The new family formulation is introduced in section 2, the new proposed distribution MGExED is presented in section 3, and some statistical properties, such as quantile function and order statistics, are demonstrated in section 4. Section 5 provides the different methods to estimate MGExED parameters. Section 6 discussed a numerical simulation for MGExED. The MGExED distribution is applied to two real datasets in section 7. Finally, section 8 provides some concluding remarks.

## 2 Family formulation

Mudholkar et al. [24] defined the exponentiated G-family with cumulative distribution function (CDF) as

(1) $$F(x;\alpha )=G^{\alpha }(x),\alpha >0,$$

and the probability density function (pdf) on the form

$$f(x;\alpha )=\alpha g(x)G^{\alpha -1}(x).$$

If we assume that the parameter *α* in the exponentiated G-family in Eq. (1) is a random variable that has a gamma distribution with parameters (*η*,*σ*), which the PDF in the form

$$h(\alpha ;\eta ,\sigma )=\frac{\alpha^{\eta -1}e^{-\frac{\alpha }{\sigma }}}{\Gamma (\eta )\sigma^{\eta }},\alpha >0,\eta >0,\sigma >0.$$

The PDF of the mixture of the gamma-exponentiated G-family is

(2) $$f_{X}(x;\eta ,\sigma )=\underset{0}{\int^{\infty }}f(x;\alpha )h(\alpha ;\eta ,\sigma )d\alpha =\frac{1}{\Gamma (\eta )}\frac{g(x)}{\sigma^{\eta }G(x)}\underset{0}{\int^{\infty }}\alpha^{\eta }e^{-\alpha \left(\frac{1}{\sigma }-\log(G(x)\right)}d\alpha =\frac{\eta \sigma g(x)}{G(x)(1-\sigma log{\left(G(x)\right)}^{\eta +1}},-\infty <x<\infty ,\eta >0,\sigma >0.$$

Lemma 1 *The function* $f_{X}(x;\eta ,\sigma )$ *in Eq.* (2) *is a PDF.*

Proof To prove $f_{X}(x;\eta ,\sigma )$ is a pdf, we need to prove that $f_{X}(x;\eta ,\sigma )\geq 0$ and $\underset{-\infty }{\int^{\infty }}f_{X}(x;\eta ,\sigma )\text{dx}=1$.

It is clear that σlog(G(x))<0 and ${\left(1-\sigma log(G(x))\right)}^{\eta +1}>0$, thus $f_{X}(x;\eta ,\sigma )\geq 0$

$$\underset{-\infty }{\int^{\infty }}f_{X}(x;\eta ,\sigma )\text{dx}={{\left(1-\sigma log(G(x))\right)}^{-\eta }|}_{-\infty }^{\infty }=1.$$

The CDF of the mixture of the gamma-exponentiated G-family mixture is

(3) $$F_{X}(x;\eta ,\sigma )=\underset{-\infty }{\int^{x}}f_{X}(x;\eta ,\sigma )dx={\left(1-\sigma log(G(x))\right)}^{-\eta }.$$

## 3 Mixture of gamma-exponentiated exponential distribution

The formulation of the mixture of gamma-exponentiated exponential distribution (MGExED) is obtained by taking the exponential distribution with scale parameter *ω* as a baseline model for Equation (3). Its CDF is defined as follows

$$F(x)={\left(1-\sigma \log\left(1-e^{-x\omega }\right)\right)}^{-\eta };x,\sigma ,\eta ,\omega >0$$

and its corresponding PDF is

(4) $$f(x)=\frac{\eta \sigma \omega e^{-x\omega }{\left(1-\sigma \log\left(1-e^{-x\omega }\right)\right)}^{-\eta -1}}{1-e^{-x\omega }}.$$

**Figure 1:**
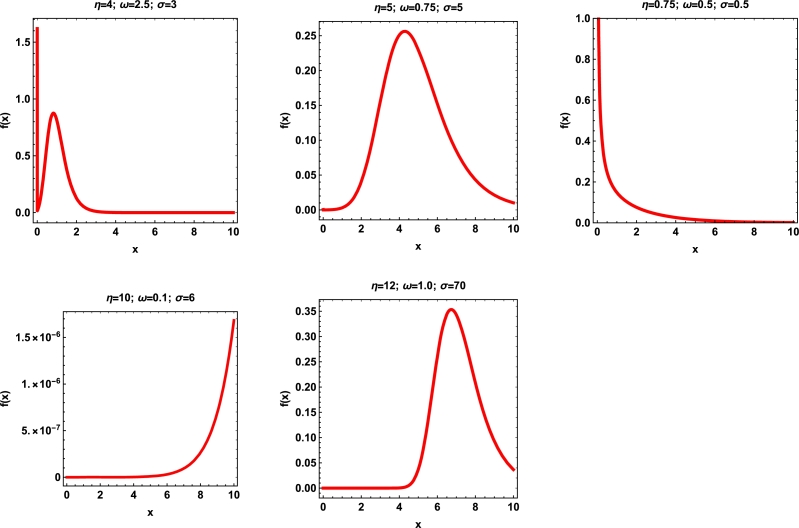
The PDF plots of the MGExED.

Some plots for PDF (4) are presented in Fig. 1, which can take several shapes for distinct values of parameters.

## 4 Statistical properties

### 4.1 Quantile function

The quantile function of the MGExED is defined as

$$QF(p)=-\frac{\log\left(1-e^{\frac{1}{\sigma }-\frac{p^{-1/\eta }}{\sigma }}\right)}{\omega }.$$

The first, second, and third quartiles of MGExED are determined by assigning *p* the values of 0.25, 0.5, and 0.75, respectively.

If *p* follows a uniform distribution between 0 and 1, the quantile function can be employed to generate a random dataset of size *n* from MGExED as follows:

$$x_{i}=-\frac{\log\left(1-e^{\frac{1}{\sigma }-\frac{p_{i}^{-1/\eta }}{\sigma }}\right)}{\omega },\;i=1,2,\ldots ,n.$$

### 4.2 Moments

Suppose *X* represents a random variable (RV) follows MGExED, the $r^{th}$ moments of *X* can be defined in the following manner

$$\mu_{r}=\eta \sigma \omega \int_{0}^{\infty }x^{r}\frac{e^{-x\omega }{\left(1-\sigma \log\left(1-e^{-x\omega }\right)\right)}^{-\eta -1}}{1-e^{-x\omega }}dx,$$

due to the complexity of obtaining an explicit form of this integral and the unavailability of a straightforward linear representation for our suggested model's PDF and CDF, evaluating this integral might necessitate numerical methods. Several software options like R, Wolfram Mathematica, and Matlab can compute this integral numerically.

### 4.3 Order statistics

If we consider a random sample of MGExED denoted by $X_{1}$, $X_{2}$,…, $X_{n}$, and their corresponding order statistics as $X_{1:n}$, $X_{2:n}$,…, $X_{n:n}$, PDF and CDF for the $i^{th}$ order statistic can be expressed as follows:

fi:n(x)=n!(i−1)!(n−i)![F(x)]i−1[1−F(x)]n−if(x)=ησωn!((1−σlog⁡(1−e−xω))−η)i(1−(1−σlog⁡(1−e−xω))−η)n−iΓ(i)(1−exω)Γ(−i+n+1)(σlog⁡(1−e−xω)−1),Fi:n(x)=∑r=in(rn)(F(x))r(1−F(x))n−r=(ni)((1−σlog⁡(1−e−xω))−η)i(1−(1−σlog⁡(1−e−xω))−η)n−iF12(1,i−n;i+1;11−(1−σlog⁡(1−e−xω))η).

## 5 Estimation methods

In the following subsections, we will use a set of parametric estimation methods to determine our proposed model estimators $\overset{ˆ}{\eta }$, $\overset{ˆ}{\sigma }$, and $\overset{ˆ}{\omega }$. For more details about these methods, see [6], [8], [17], [34], [18], [25], [35], [3].

### 5.1 Method of maximum likelihood

The maximum likelihood method ($EM_{1}$) is a conventional statistical estimation technique. Maximum likelihood estimators (MLEs) are favored for their advantageous properties, offering the means to derive confidence intervals for model parameters. In sufficiently large sample sizes, the normal approximation for these estimators can be conveniently managed through analytical or numerical methods. Our initial analysis estimates the proposed model parameters, employing the maximum likelihood approach, particularly for complete samples. The log-likelihood function for our proposed model is defined as follows:

$$\log L=-(\eta +1)\sum_{i=1}^{n}\log\left(\sigma \omega x_{i}-\sigma \log\left(e^{\omega x_{i}}-1\right)+1\right)-\sum_{i=1}^{n}\log\left(1-e^{\omega x_{i}}\right)+n\log(\eta \sigma \omega ).$$

### 5.2 Method of Anderson Darling

It was first presented as a substitute for conventional statistical techniques for identifying sample distribution deviations from the predicted distribution. The Anderson-Darling Estimates ($EM_{2}$) for the proposed distribution can be obtained by optimizing the following function concerning the model's parameters:

$$A(x_{i})=-n-\frac{1}{n}\sum_{i=1}^{n}(2i-1)[\log F(x_{i:n})+\log S(x_{n-i-1:n})]=-n-\frac{1}{n}\sum_{i=1}^{n}(2i-1)\left[-\eta \log\left(1-\sigma \log\left(1-e^{\omega \left(-x_{i:n}\right)}\right)\right)+\log\left(1-{\left(1-\sigma \log\left(1-e^{\omega \left(-x_{i:n}\right)}\right)\right)}^{-\eta }\right)\right].$$

### 5.3 Method of Cramer von Mises

The Cramér von Mises estimators ($EM_{3}$) are obtained by minimizing the disparity between the cumulative distribution function (CDF) and the empirical distribution. This part $\frac{2i-1}{2n}$ represents the empirical estimation of the CDF at $x_{i:n}$, which is computed from a sample $x_{i:n}$. The estimates are determined numerically by optimizing the following function:

$$C(x_{i})=\frac{1}{12n}+\sum_{i=1}^{n}{\left[F(x_{i:n})-\frac{2i-1}{2n}\right]}^{2}=\frac{1}{12n}+\sum_{i=1}^{n}{\left[{\left(1-\sigma \log\left(1-e^{\omega \left(-x_{i:n}\right)}\right)\right)}^{-\eta }-\frac{2i-1}{2n}\right]}^{2}.$$

### 5.4 Method of maximum product of spacings

When the MLE estimates do not hold up, the maximum product of spacings method offers a strong substitute for the MLE. The geometric mean of the spacings between the CDFs of closely spaced observations is maximized using this strategy. The maximum product of spacings estimators ($EM_{4}$) for the proposed distribution can be derived by maximizing the following function for the model's parameters.

$$\delta \left(x_{i}\right)=\frac{1}{n+1}\sum_{i=1}^{n+1}\log\varpi_{i}(x_{i}),$$

where $\varpi_{i}(x_{i})=F(x_{i:n})-F(x_{i-1:n})$, $F(x_{0:n})=0$ and $F(x_{n+1:n})=1$.

### 5.5 Methods of least squares

The proposed distribution's least squares estimates ($EM_{5}$) can be calculated by minimizing the subsequent function about the model's parameter.

$$V(x_{i})=\sum_{i=1}^{n}{\left[F(x_{i:n})-\frac{i}{n+1}\right]}^{2}=\sum_{i=1}^{n}{\left[{\left(1-\sigma \log\left(1-e^{\omega \left(-x_{i:n}\right)}\right)\right)}^{-\eta }-\frac{i}{n+1}\right]}^{2}.$$

### 5.6 Methods of right tail Anderson Darling

Targeting the right tail of the distribution's CDF, a variation of the Anderson-Darling statistic is optimized in the RTADT. This method improves parameter estimate accuracy by concentrating on the traits and behavior of the distribution's right tail. The right tail Anderson-Darling estimators ($EM_{6}$) for the parameters of the proposed distribution can be acquired through the minimization of the following function.

$$R(x_{i})=\frac{n}{2}-2\sum_{i=1}^{n}F(x_{i:n})-\frac{1}{n}\sum_{i=1}^{n}(2i-1)\log S(x_{i:n})=\frac{n}{2}-2\sum_{i=1}^{n}{\left(1-\sigma \log\left(1-e^{\omega \left(-x_{i:n}\right)}\right)\right)}^{-\eta }-\frac{1}{n}\sum_{i=1}^{n}(2i-1)\log\left(1-{\left(1-\sigma \log\left(1-e^{\omega \left(-x_{i:n}\right)}\right)\right)}^{-\eta }\right).$$

### 5.7 Methods of weighted least squares

Estimating parameters within the proposed distribution can be accomplished using the weighted least squares method denoted as $EM_{7}$. This estimation approach entails the minimization of the subsequent function:

$$W(x_{i})=\sum_{i=1}^{n}\frac{{\left(n+1\right)}^{2}(n+2)}{i(n-i+1)}{\left[F(x_{i:n})-\frac{i}{n+1}\right]}^{2}=\sum_{i=1}^{n}\frac{{\left(n+1\right)}^{2}(n+2)}{i(n-i+1)}{\left[{\left(1-\sigma \log\left(1-e^{\omega \left(-x_{i:n}\right)}\right)\right)}^{-\eta }-\frac{i}{n+1}\right]}^{2}.$$

### 5.8 Methods of left tail Anderson Darling

The model parameters' left tail Anderson-Darling estimates ($EM_{8}$) can be derived by minimizing the subsequent function.

$$L(x_{i})=-\frac{3}{2}n+2\sum_{i=1}^{n}F(x_{i:n})-\frac{1}{n}\sum_{i=1}^{n}(2i-1)\log F(x_{i:n})=-\frac{3}{2}n+2\sum_{i=1}^{n}F(x_{i:n})+\frac{1}{n}\sum_{i=1}^{n}(2i-1)\eta \log\left(1-\sigma \log\left(1-e^{\omega \left(-x_{i:n}\right)}\right)\right).$$

### 5.9 Minimum spacing absolute distance

This method is useful for parameter estimation because of its ease of use and capacity to calculate the separations between data points. The minimum spacing absolute distance estimates ($EM_{9}$) can be obtained by minimizing the function:

$$\zeta \left(x_{i}\right)=\sum_{i=1}^{n+1}|\varpi_{i}-\frac{1}{n+1}|.$$

### 5.10 Minimum spacing absolute-log distance

By using the logarithm of the distances, this method improves the minimum spacing strategy and is useful for managing data sets with varying sizes. The minimum spacing absolute-log distance estimates ($EM_{10}$) of the parameters proposed are obtained by optimizing the following function.

$${ϒ}_{2}\left(x_{i}\right)=\sum_{i=1}^{n+1}|\log\varpi_{i}-\log\frac{1}{n+1}|.$$

### 5.11 Anderson Darling left tail second order

The Anderson-Darling left-tail second-order estimators ($EM_{11}$) for the parameters of the proposed distribution can be acquired through the minimization of the following function.

$$LTS=2\sum_{i=1}^{n}\log F(x_{i})+\frac{1}{n}\sum_{i=1}^{n}\frac{\left(2i-1\right)}{F(x_{i})}=-2\sum_{i=1}^{n}\eta \log\left(1-\sigma \log\left(1-e^{\omega \left(-x_{i:n}\right)}\right)\right)+\frac{1}{n}\sum_{i=1}^{n}\frac{\left(2i-1\right)}{{\left(1-\sigma \log\left(1-e^{\omega \left(-x_{i:n}\right)}\right)\right)}^{-\eta }}.$$

### 5.12 Method of Kolmogorov

The Kolmogorov estimators ($EM_{12}$) for the parameters of the proposed distribution can be obtained through the optimization of the following function.

$$KM=\underset{1\leq i\leq n}{MAX}\left[\frac{i}{n}-F(x_{i}),F(x_{i})-\frac{i-1}{n}\right]=\underset{1\leq i\leq n}{MAX}\left[\frac{i}{n}-{\left(1-\sigma \log\left(1-e^{\omega \left(-x_{i:n}\right)}\right)\right)}^{-\eta },{\left(1-\sigma \log\left(1-e^{\omega \left(-x_{i:n}\right)}\right)\right)}^{-\eta }-\frac{i-1}{n}\right].$$

## 6 Numerical simulation

In this section, we will apply the range of estimation methods previously delineated to identify estimators for our proposed model using randomly generated datasets. The primary aim of this investigation is to comprehensively scrutinize the performance and behavior of these estimation techniques. To assess the efficacy of these methods, we will employ a suite of metrics, including the average absolute bias (BIAS), mean square error (MSE), and mean relative error (MRE). These measures will be systematically calculated for varying parameter configurations and sample sizes, employing the R programming language. We will execute a simulation study to ascertain our model's most precise method for parameter estimation. This simulation will involve the generation of five thousand random samples, each with differing sample sizes (20, 80, 150, 250, 400, and 500).

The results of the numerical simulations have been documented in Table 1, Table 2, Table 3, Table 4, Table 5. These tables provide insight into the power of each technique compared to others. The tables also include partial and total ranking of our estimators, as summarized in Table 6. Upon scrutinizing the simulation outcomes and the ranking table, it is evident that the estimators proposed for our model exhibit properties of consistency. As the sample size increases, a consistent trend is observed where most of the performance measures in the simulation tables decrease. The ranking table shows that the Kolmogorov estimation method is the most favored among the various techniques, indicating its strong performance and suitability for the model parameter estimation.

**Table 1:** Numerical values of simulation measures (BIAS, MSE, and MRE) for *η* = 2.5, *σ* = 1.5, *ω* = 0.75.

n	Est.	Est.Par.	*EM*_1_	*EM*_2_	*EM*_3_	*EM*_4_	*EM*_5_	*EM*_6_	*EM*_7_	*EM*_8_	*EM*_8_	*EM*_10_	*EM*_11_	*EM*_12_
20	BIAS	$\overset{ˆ}{\eta }$	1.05075^{4}^	1.05384^{5}^	1.07341^{9}^	1.09331^{11}^	1.09006^{10}^	1.06453^{6}^	1.06804^{7}^	1.0734^{8}^	0.87338^{2}^	0.93975^{3}^	1.10846^{12}^	0.44796^{1}^
		$\overset{ˆ}{\sigma }$	0.47724^{10}^	0.46443^{7}^	0.47609^{9}^	0.44363^{2}^	0.46577^{8}^	0.48433^{12}^	0.45154^{4}^	0.45695^{6}^	0.44902^{3}^	0.45433^{5}^	0.48305^{11}^	0.37749^{1}^
		$\overset{ˆ}{\omega }$	0.13212^{2}^	0.13616^{4}^	0.14154^{5}^	0.15201^{8}^	0.15619^{11}^	0.1345^{3}^	0.1467^{6}^	0.14925^{7}^	0.15226^{9}^	0.15391^{10}^	0.17003^{12}^	0.12854^{1}^
	MSE	$\overset{ˆ}{\eta }$	1.48238^{4}^	1.50877^{5}^	1.55302^{9}^	1.58539^{10}^	1.5998^{11}^	1.52843^{6}^	1.53282^{7}^	1.55194^{8}^	1.15881^{2}^	1.28908^{3}^	1.64298^{12}^	0.37769^{1}^
		$\overset{ˆ}{\sigma }$	0.36833^{10}^	0.34739^{8}^	0.35657^{9}^	0.30731^{2}^	0.33504^{6}^	0.37025^{11}^	0.32269^{5}^	0.33913^{7}^	0.31471^{3}^	0.3198^{4}^	0.37102^{12}^	0.23925^{1}^
		$\overset{ˆ}{\omega }$	0.02745^{1}^	0.02915^{4}^	0.0318^{5}^	0.03342^{7}^	0.03574^{11}^	0.02852^{3}^	0.03218^{6}^	0.03514^{9}^	0.0352^{10}^	0.03467^{8}^	0.04473^{12}^	0.02812^{2}^
	MRE	$\overset{ˆ}{\eta }$	0.4203^{3}^	0.42154^{4}^	0.42937^{8}^	0.43732^{10}^	0.43603^{9}^	0.42581^{5}^	0.42722^{6}^	0.42936^{7}^	0.34935^{1}^	0.3759^{2}^	0.44338^{11.5}^	0.44338^{11.5}^
		$\overset{ˆ}{\sigma }$	0.31816^{10}^	0.30962^{7}^	0.31739^{9}^	0.29575^{2}^	0.31051^{8}^	0.32289^{11}^	0.30103^{4}^	0.30464^{6}^	0.29935^{3}^	0.30289^{5}^	1.6176^{12}^	0.25166^{1}^
		$\overset{ˆ}{\omega }$	0.17616^{2}^	0.18155^{4}^	0.18872^{5}^	0.20268^{8}^	0.20825^{11}^	0.17933^{3}^	0.1956^{6}^	0.199^{7}^	0.20301^{9}^	0.20521^{10}^	0.78796^{12}^	0.17139^{1}^
	∑*Ranks*		61^{3}^	63^{4}^	90^{10}^	80^{8}^	113^{11}^	79^{7}^	67^{5.5}^	85^{9}^	55^{2}^	67^{5.5}^	142^{12}^	34^{1}^

80	BIAS	$\overset{ˆ}{\eta }$	0.92282^{3}^	0.94836^{6}^	0.99622^{11}^	0.94555^{5}^	0.9952^{9}^	0.99541^{10}^	0.96751^{7}^	0.98247^{8}^	0.86993^{2}^	0.93942^{4}^	1.0392^{12}^	0.38976^{1}^
		$\overset{ˆ}{\sigma }$	0.38332^{5}^	0.38791^{6}^	0.41329^{11}^	0.35187^{2}^	0.41058^{10}^	0.43418^{12}^	0.39038^{7}^	0.39193^{8}^	0.36015^{3}^	0.37361^{4}^	0.40929^{9}^	0.23108^{1}^
		$\overset{ˆ}{\omega }$	0.07814^{2}^	0.07953^{4}^	0.08373^{7}^	0.08019^{6}^	0.08499^{8}^	0.0793^{3}^	0.08003^{5}^	0.08785^{10}^	0.08879^{11}^	0.08701^{9}^	0.11117^{12}^	0.06947^{1}^
	MSE	$\overset{ˆ}{\eta }$	1.19065^{3}^	1.24331^{4}^	1.3652^{11}^	1.25758^{6}^	1.35708^{10}^	1.35084^{9}^	1.29535^{7}^	1.31356^{8}^	1.13826^{2}^	1.25104^{5}^	1.45404^{12}^	0.29727^{1}^
		$\overset{ˆ}{\sigma }$	0.26618^{6}^	0.26199^{5}^	0.29194^{10}^	0.2185^{2}^	0.28787^{9}^	0.32084^{12}^	0.27046^{7}^	0.27299^{8}^	0.22742^{3}^	0.24375^{4}^	0.29634^{11}^	0.09852^{1}^
		$\overset{ˆ}{\omega }$	0.00955^{2}^	0.0096^{3}^	0.01097^{7}^	0.00979^{4}^	0.01115^{8}^	0.00982^{5}^	0.00986^{6}^	0.01186^{10}^	0.01224^{11}^	0.01161^{9}^	0.01891^{12}^	0.00782^{1}^
	MRE	$\overset{ˆ}{\eta }$	0.36913^{2}^	0.37934^{5}^	0.39849^{10}^	0.37822^{4}^	0.39808^{8}^	0.39816^{9}^	0.387^{6}^	0.39299^{7}^	0.34797^{1}^	0.37577^{3}^	0.41568^{11.5}^	0.41568^{11.5}^
		$\overset{ˆ}{\sigma }$	0.25555^{5}^	0.25861^{6}^	0.27552^{10}^	0.23458^{2}^	0.27372^{9}^	0.28945^{11}^	0.26025^{7}^	0.26129^{8}^	0.2401^{3}^	0.24907^{4}^	1.55092^{12}^	0.15405^{1}^
		$\overset{ˆ}{\omega }$	0.10418^{2}^	0.10604^{4}^	0.11164^{7}^	0.10692^{6}^	0.11333^{8}^	0.10574^{3}^	0.1067^{5}^	0.11713^{10}^	0.11839^{11}^	0.11601^{9}^	0.76643^{12}^	0.09262^{1}^
	∑*Ranks*		39^{2}^	58^{4}^	111^{11}^	49^{3}^	104^{10}^	97^{8}^	75^{7}^	102^{9}^	62^{5}^	67^{6}^	139^{12}^	33^{1}^

150	BIAS	$\overset{ˆ}{\eta }$	0.85437^{4}^	0.89673^{6}^	0.93896^{9}^	0.84108^{3}^	0.94639^{10}^	0.94919^{11}^	0.898^{7}^	0.90537^{8}^	0.83392^{2}^	0.87074^{5}^	0.97802^{12}^	0.35878^{1}^
		$\overset{ˆ}{\sigma }$	0.34511^{8}^	0.3449^{7}^	0.38245^{11}^	0.30238^{2}^	0.37912^{10}^	0.40797^{12}^	0.34033^{5}^	0.34413^{6}^	0.31668^{4}^	0.315^{3}^	0.36781^{9}^	0.17473^{1}^
		$\overset{ˆ}{\omega }$	0.06195^{2}^	0.06483^{6}^	0.06719^{7}^	0.06347^{4}^	0.0687^{8}^	0.06298^{3}^	0.06384^{5}^	0.0725^{11}^	0.07159^{10}^	0.06908^{9}^	0.09726^{12}^	0.05097^{1}^
	MSE	$\overset{ˆ}{\eta }$	1.0357^{3}^	1.13869^{6}^	1.2252^{9}^	1.02501^{2}^	1.25414^{11}^	1.24099^{10}^	1.15014^{7}^	1.16817^{8}^	1.05428^{4}^	1.11708^{5}^	1.3147^{12}^	0.25179^{1}^
		$\overset{ˆ}{\sigma }$	0.21865^{8}^	0.21673^{6}^	0.25663^{11}^	0.16857^{2}^	0.25568^{10}^	0.28558^{12}^	0.209^{5}^	0.21816^{7}^	0.18291^{4}^	0.18206^{3}^	0.2499^{9}^	0.05596^{1}^
		$\overset{ˆ}{\omega }$	0.00587^{2}^	0.00643^{6}^	0.00699^{7}^	0.00622^{4.5}^	0.00721^{8}^	0.00609^{3}^	0.00622^{4.5}^	0.00806^{11}^	0.00789^{10}^	0.00737^{9}^	0.01432^{12}^	0.0042^{1}^
	MRE	$\overset{ˆ}{\eta }$	0.34175^{3}^	0.35869^{5}^	0.37558^{8}^	0.33643^{2}^	0.37856^{9}^	0.37967^{10}^	0.3592^{6}^	0.36215^{7}^	0.33357^{1}^	0.3483^{4}^	0.39121^{11.5}^	0.39121^{11.5}^
		$\overset{ˆ}{\sigma }$	0.23007^{8}^	0.22993^{7}^	0.25497^{10}^	0.20159^{2}^	0.25275^{9}^	0.27198^{11}^	0.22688^{5}^	0.22942^{6}^	0.21112^{4}^	0.21^{3}^	1.52858^{12}^	0.11649^{1}^
		$\overset{ˆ}{\omega }$	0.08261^{2}^	0.08644^{6}^	0.08959^{7}^	0.08462^{4}^	0.0916^{8}^	0.08398^{3}^	0.08512^{5}^	0.09667^{11}^	0.09546^{10}^	0.09211^{9}^	0.76058^{12}^	0.06796^{1}^
	∑*Ranks*		53^{3}^	73^{7}^	104^{10}^	33.5^{2}^	109^{11}^	99^{8.5}^	65.5^{5}^	99^{8.5}^	64^{4}^	66^{6}^	137^{12}^	33^{1}^
250	BIAS	$\overset{ˆ}{\eta }$	0.7391^{3}^	0.80896^{6}^	0.88136^{9}^	0.72279^{2}^	0.89152^{10}^	0.89983^{12}^	0.81143^{7}^	0.8311^{8}^	0.7823^{5}^	0.77948^{4}^	0.89478^{11}^	0.34957^{1}^
		$\overset{ˆ}{\sigma }$	0.28008^{5}^	0.29607^{8}^	0.3359^{11}^	0.24538^{2}^	0.3315^{10}^	0.37061^{12}^	0.28491^{6}^	0.29542^{7}^	0.27607^{4}^	0.27585^{3}^	0.31777^{9}^	0.14405^{1}^
		$\overset{ˆ}{\omega }$	0.05111^{2}^	0.05371^{6}^	0.05779^{8}^	0.05184^{3}^	0.05841^{9}^	0.05318^{4}^	0.05362^{5}^	0.06189^{11}^	0.05965^{10}^	0.05765^{7}^	0.08406^{12}^	0.04141^{1}^
	MSE	$\overset{ˆ}{\eta }$	0.80716^{3}^	0.96782^{6}^	1.10606^{9}^	0.78609^{2}^	1.13896^{11}^	1.14657^{12}^	0.98129^{7}^	1.00722^{8}^	0.93548^{5}^	0.91451^{4}^	1.13696^{10}^	0.23989^{1}^
		$\overset{ˆ}{\sigma }$	0.14629^{5}^	0.16203^{7}^	0.20209^{11}^	0.11357^{2}^	0.19903^{10}^	0.24604^{12}^	0.14981^{6}^	0.16274^{8}^	0.14256^{3}^	0.14329^{4}^	0.1988^{9}^	0.03828^{1}^
		$\overset{ˆ}{\omega }$	0.00403^{2}^	0.00444^{6}^	0.00507^{7}^	0.00409^{3}^	0.00523^{9}^	0.00431^{4}^	0.00443^{5}^	0.00588^{11}^	0.00551^{10}^	0.00508^{8}^	0.01075^{12}^	0.00271^{1}^
	MRE	$\overset{ˆ}{\eta }$	0.29564^{2}^	0.32358^{5}^	0.35254^{8}^	0.28912^{1}^	0.35661^{9}^	0.35993^{12}^	0.32457^{6}^	0.33244^{7}^	0.31292^{4}^	0.31179^{3}^	0.35791^{10.5}^	0.35791^{10.5}^
		$\overset{ˆ}{\sigma }$	0.18672^{5}^	0.19738^{8}^	0.22393^{10}^	0.16359^{2}^	0.221^{9}^	0.24708^{11}^	0.18994^{6}^	0.19694^{7}^	0.18405^{4}^	0.1839^{3}^	1.51335^{12}^	0.09603^{1}^
		$\overset{ˆ}{\omega }$	0.06815^{2}^	0.07162^{6}^	0.07705^{8}^	0.06912^{3}^	0.07788^{9}^	0.07091^{4}^	0.0715^{5}^	0.08252^{11}^	0.07953^{10}^	0.07686^{7}^	0.75642^{12}^	0.05521^{1}^
	∑*Ranks*		38^{3}^	77^{7}^	107^{9}^	26^{1}^	113^{11}^	110^{10}^	70^{5}^	103^{8}^	73^{6}^	56^{4}^	132^{12}^	31^{2}^

400	BIAS	$\overset{ˆ}{\eta }$	0.626^{3}^	0.70504^{6}^	0.8038^{9}^	0.61047^{2}^	0.80934^{10}^	0.83615^{12}^	0.72628^{7}^	0.72942^{8}^	0.69671^{5}^	0.67772^{4}^	0.80943^{11}^	0.33272^{1}^
		$\overset{ˆ}{\sigma }$	0.22726^{3}^	0.24582^{7}^	0.29386^{11}^	0.20103^{2}^	0.28748^{9}^	0.32559^{12}^	0.24071^{6}^	0.2502^{8}^	0.23235^{5}^	0.22901^{4}^	0.2885^{10}^	0.1259^{1}^
		$\overset{ˆ}{\omega }$	0.04242^{3}^	0.0453^{5}^	0.04846^{8}^	0.04172^{2}^	0.05008^{9}^	0.0461^{6}^	0.04491^{4}^	0.0516^{11}^	0.05126^{10}^	0.04835^{7}^	0.07614^{12}^	0.03424^{1}^
	MSE	$\overset{ˆ}{\eta }$	0.59227^{3}^	0.76527^{6}^	0.95892^{10}^	0.58048^{2}^	0.97773^{11}^	1.02602^{12}^	0.81151^{8}^	0.80335^{7}^	0.75638^{5}^	0.70173^{4}^	0.92228^{9}^	0.21552^{1}^
		$\overset{ˆ}{\sigma }$	0.09475^{3}^	0.11123^{7}^	0.15605^{10}^	0.07289^{2}^	0.14975^{9}^	0.19063^{12}^	0.10514^{5}^	0.11677^{8}^	0.10588^{6}^	0.09777^{4}^	0.15783^{11}^	0.02948^{1}^
		$\overset{ˆ}{\omega }$	0.00278^{3}^	0.00316^{5}^	0.00361^{8}^	0.00271^{2}^	0.00379^{9}^	0.00324^{6}^	0.00312^{4}^	0.00407^{11}^	0.00404^{10}^	0.00359^{7}^	0.00867^{12}^	0.00188^{1}^
	MRE	$\overset{ˆ}{\eta }$	0.2504^{2}^	0.28201^{5}^	0.32152^{8}^	0.24419^{1}^	0.32374^{9}^	0.33446^{12}^	0.29051^{6}^	0.29177^{7}^	0.27869^{4}^	0.27109^{3}^	0.32377^{10.5}^	0.32377^{10.5}^
		$\overset{ˆ}{\sigma }$	0.15151^{3}^	0.16388^{7}^	0.1959^{10}^	0.13402^{2}^	0.19166^{9}^	0.21706^{11}^	0.16047^{6}^	0.1668^{8}^	0.1549^{5}^	0.15268^{4}^	1.50763^{12}^	0.08394^{1}^
		$\overset{ˆ}{\omega }$	0.05655^{3}^	0.0604^{5}^	0.06462^{8}^	0.05563^{2}^	0.06678^{9}^	0.06147^{6}^	0.05988^{4}^	0.0688^{11}^	0.06834^{10}^	0.06446^{7}^	0.75482^{12}^	0.04565^{1}^
	∑*Ranks*		34^{3}^	70^{6}^	108^{9}^	22^{1}^	111^{10}^	118^{11}^	66^{5}^	105^{8}^	79^{7}^	58^{4}^	134^{12}^	31^{2}^

500	BIAS	$\overset{ˆ}{\eta }$	0.56454^{3}^	0.6575^{6}^	0.76032^{9}^	0.55689^{2}^	0.77537^{11}^	0.79424^{12}^	0.67373^{7}^	0.67708^{8}^	0.63581^{5}^	0.61395^{4}^	0.76637^{10}^	0.3188^{1}^
		$\overset{ˆ}{\sigma }$	0.20716^{4}^	0.21731^{7}^	0.27201^{9}^	0.18516^{2}^	0.27302^{10}^	0.30667^{12}^	0.21528^{6}^	0.22745^{8}^	0.20778^{5}^	0.20683^{3}^	0.2749^{11}^	0.11948^{1}^
		$\overset{ˆ}{\omega }$	0.03849^{3}^	0.04141^{5}^	0.04659^{8}^	0.03833^{2}^	0.04667^{9}^	0.04282^{6}^	0.04067^{4}^	0.04732^{11}^	0.04696^{10}^	0.04371^{7}^	0.07109^{12}^	0.0315^{1}^
	MSE	$\overset{ˆ}{\eta }$	0.4946^{3}^	0.68397^{6}^	0.86433^{10}^	0.48473^{2}^	0.89617^{11}^	0.94278^{12}^	0.71238^{8}^	0.7025^{7}^	0.63747^{5}^	0.57811^{4}^	0.84551^{9}^	0.20357^{1}^
		$\overset{ˆ}{\sigma }$	0.07957^{3}^	0.08567^{7}^	0.13575^{10}^	0.06233^{2}^	0.13252^{9}^	0.1716^{12}^	0.08165^{5}^	0.09506^{8}^	0.0834^{6}^	0.07984^{4}^	0.14312^{11}^	0.02596^{1}^
		$\overset{ˆ}{\omega }$	0.0023^{3}^	0.00265^{5}^	0.00328^{8}^	0.00228^{2}^	0.00331^{9}^	0.00278^{6}^	0.00257^{4}^	0.00344^{11}^	0.00341^{10}^	0.00298^{7}^	0.00768^{12}^	0.00157^{1}^
	MRE	$\overset{ˆ}{\eta }$	0.22582^{2}^	0.263^{5}^	0.30413^{8}^	0.22276^{1}^	0.31015^{11}^	0.3177^{12}^	0.26949^{6}^	0.27083^{7}^	0.25432^{4}^	0.24558^{3}^	0.30655^{9.5}^	0.30655^{9.5}^
		$\overset{ˆ}{\sigma }$	0.1381^{4}^	0.14487^{7}^	0.18134^{9}^	0.12344^{2}^	0.18202^{10}^	0.20445^{11}^	0.14352^{6}^	0.15163^{8}^	0.13852^{5}^	0.13789^{3}^	1.50637^{12}^	0.07965^{1}^
		$\overset{ˆ}{\omega }$	0.05132^{3}^	0.05521^{5}^	0.06212^{8}^	0.05111^{2}^	0.06223^{9}^	0.05709^{6}^	0.05422^{4}^	0.06309^{11}^	0.06261^{10}^	0.05828^{7}^	0.75539^{12}^	0.042^{1}^
	∑*Ranks*		37^{3}^	70^{6}^	104^{8}^	22^{1}^	119^{11}^	118^{10}^	66^{5}^	105^{9}^	79^{7}^	55^{4}^	132^{12}^	29^{2}^

**Table 2:** Numerical values of simulation measures (BIAS, MSE, and MRE) for *η* = 1.5, *σ* = 2.5, *ω* = 0.9.

n	Est.	Est.Par.	*EM*_1_	*EM*_2_	*EM*_3_	*EM*_4_	*EM*_5_	*EM*_6_	*EM*_7_	*EM*_8_	*EM*_8_	*EM*_10_	*EM*_11_	*EM*_12_
20	BIAS	$\overset{ˆ}{\eta }$	0.61445^{4}^	0.62394^{7}^	0.61935^{6}^	0.6329^{10}^	0.63479^{11}^	0.6293^{8}^	0.63071^{9}^	0.61918^{5}^	0.58868^{2}^	0.6003^{3}^	0.64797^{12}^	0.41299^{1}^
		$\overset{ˆ}{\sigma }$	0.79942^{6}^	0.79772^{5}^	0.80814^{9}^	0.80026^{7}^	0.81135^{10}^	0.81868^{12}^	0.78832^{4}^	0.78443^{3}^	0.78306^{2}^	0.81171^{11}^	0.80539^{8}^	0.47331^{1}^
		$\overset{ˆ}{\omega }$	0.15266^{1}^	0.15726^{2}^	0.16746^{5}^	0.17431^{8}^	0.17627^{9}^	0.163^{3}^	0.16873^{6}^	0.16997^{7}^	0.1862^{11}^	0.18299^{10}^	0.18628^{12}^	0.16557^{4}^
	MSE	$\overset{ˆ}{\eta }$	0.51543^{4}^	0.53503^{7}^	0.52921^{6}^	0.5419^{9}^	0.54681^{11}^	0.53794^{8}^	0.54308^{10}^	0.52642^{5}^	0.4813^{2}^	0.49768^{3}^	0.56366^{12}^	0.27931^{1}^
		$\overset{ˆ}{\sigma }$	1.01866^{12}^	0.98294^{6}^	1.00596^{9}^	0.93637^{2}^	0.98311^{7}^	1.01776^{11}^	0.93754^{3}^	0.96809^{5}^	0.93863^{4}^	0.98711^{8}^	1.01485^{10}^	0.4277^{1}^
		$\overset{ˆ}{\omega }$	0.03707^{1}^	0.03847^{2}^	0.04407^{6}^	0.04389^{5}^	0.046^{8}^	0.04113^{3}^	0.04218^{4}^	0.04458^{7}^	0.05195^{11}^	0.05006^{10}^	0.05373^{12}^	0.0466^{9}^
	MRE	$\overset{ˆ}{\eta }$	0.40963^{3}^	0.41596^{6}^	0.4129^{5}^	0.42193^{9}^	0.42319^{10}^	0.41954^{7}^	0.42047^{8}^	0.41279^{4}^	0.39245^{1}^	0.4002^{2}^	0.43198^{11.5}^	0.43198^{11.5}^
		$\overset{ˆ}{\sigma }$	0.31977^{6}^	0.31909^{5}^	0.32326^{8}^	0.32011^{7}^	0.32454^{9}^	0.32747^{11}^	0.31533^{4}^	0.31377^{3}^	0.31322^{2}^	0.32468^{10}^	2.71408^{12}^	0.18932^{1}^
		$\overset{ˆ}{\omega }$	0.16963^{1}^	0.17473^{2}^	0.18607^{5}^	0.19367^{8}^	0.19585^{9}^	0.18111^{3}^	0.18748^{6}^	0.18885^{7}^	0.20689^{11}^	0.20332^{10}^	0.94284^{12}^	0.18396^{4}^
	∑*Ranks*		48^{1}^	55^{3}^	77^{7}^	89^{9.5}^	112^{11}^	87^{8}^	72^{6}^	60^{4.5}^	60^{4.5}^	89^{9.5}^	137^{12}^	50^{2}^

80	BIAS	$\overset{ˆ}{\eta }$	0.55944^{3}^	0.57235^{6}^	0.58632^{10}^	0.56637^{4}^	0.5964^{11}^	0.5712^{5}^	0.57299^{7}^	0.57763^{8}^	0.54532^{2}^	0.58225^{9}^	0.60777^{12}^	0.28678^{1}^
		$\overset{ˆ}{\sigma }$	0.70773^{7}^	0.71123^{9}^	0.73901^{12}^	0.66866^{3}^	0.71871^{10}^	0.73358^{11}^	0.69866^{5}^	0.70198^{6}^	0.66458^{2}^	0.6739^{4}^	0.70779^{8}^	0.32022^{1}^
		$\overset{ˆ}{\omega }$	0.08665^{2}^	0.08845^{4}^	0.09425^{7}^	0.0896^{6}^	0.09578^{8}^	0.08765^{3}^	0.08868^{5}^	0.09876^{9}^	0.10269^{11}^	0.09986^{10}^	0.11726^{12}^	0.0861^{1}^
	MSE	$\overset{ˆ}{\eta }$	0.4399^{3}^	0.45624^{5}^	0.47968^{10}^	0.45135^{4}^	0.49408^{11}^	0.46327^{7}^	0.45819^{6}^	0.46734^{8}^	0.4297^{2}^	0.47302^{9}^	0.50489^{12}^	0.14612^{1}^
		$\overset{ˆ}{\sigma }$	0.84281^{9}^	0.84225^{8}^	0.89753^{12}^	0.71884^{2}^	0.83363^{7}^	0.86834^{11}^	0.80462^{5}^	0.8285^{6}^	0.73613^{3}^	0.74456^{4}^	0.84373^{10}^	0.19396^{1}^
		$\overset{ˆ}{\omega }$	0.01166^{1}^	0.01212^{3}^	0.01398^{7}^	0.01232^{6}^	0.01432^{8}^	0.01222^{5}^	0.01208^{2}^	0.01516^{9}^	0.01646^{11}^	0.0155^{10}^	0.0214^{12}^	0.0122^{4}^
	MRE	$\overset{ˆ}{\eta }$	0.37296^{2}^	0.38156^{5}^	0.39088^{9}^	0.37758^{3}^	0.3976^{10}^	0.3808^{4}^	0.38199^{6}^	0.38509^{7}^	0.36354^{1}^	0.38817^{8}^	0.40518^{11.5}^	0.40518^{11.5}^
		$\overset{ˆ}{\sigma }$	0.28309^{7}^	0.28449^{8}^	0.2956^{11}^	0.26746^{3}^	0.28748^{9}^	0.29343^{10}^	0.27946^{5}^	0.28079^{6}^	0.26583^{2}^	0.26956^{4}^	2.60476^{12}^	0.12809^{1}^
		$\overset{ˆ}{\omega }$	0.09628^{2}^	0.09828^{4}^	0.10473^{7}^	0.09956^{6}^	0.10642^{8}^	0.09739^{3}^	0.09853^{5}^	0.10973^{9}^	0.1141^{11}^	0.11095^{10}^	0.91587^{12}^	0.09567^{1}^
	∑*Ranks*		47^{2}^	69^{6}^	112^{11}^	49^{3}^	109^{10}^	76^{7}^	62^{5}^	90^{8.5}^	59^{4}^	90^{8.5}^	137^{12}^	36^{1}^

150	BIAS	$\overset{ˆ}{\eta }$	0.53078^{2}^	0.54554^{6}^	0.5575^{9}^	0.54141^{4}^	0.56739^{11}^	0.56468^{10}^	0.54709^{7}^	0.55028^{8}^	0.53297^{3}^	0.54291^{5}^	0.58644^{12}^	0.23915^{1}^
		$\overset{ˆ}{\sigma }$	0.65304^{6}^	0.66011^{8}^	0.6986^{11}^	0.60798^{3}^	0.6846^{10}^	0.71669^{12}^	0.65756^{7}^	0.64703^{5}^	0.60641^{2}^	0.60945^{4}^	0.66812^{9}^	0.26981^{1}^
		$\overset{ˆ}{\omega }$	0.06783^{2}^	0.07036^{6}^	0.07369^{7}^	0.06892^{4}^	0.07453^{8}^	0.06869^{3}^	0.06953^{5}^	0.07694^{10}^	0.07891^{11}^	0.0766^{9}^	0.09913^{12}^	0.06458^{1}^
	MSE	$\overset{ˆ}{\eta }$	0.40047^{2}^	0.42455^{5}^	0.43887^{9}^	0.42439^{4}^	0.45675^{11}^	0.45432^{10}^	0.42586^{6}^	0.43138^{8}^	0.41624^{3}^	0.42885^{7}^	0.47619^{12}^	0.10451^{1}^
		$\overset{ˆ}{\sigma }$	0.74311^{7}^	0.74606^{8}^	0.82105^{11}^	0.62084^{2}^	0.77313^{10}^	0.84863^{12}^	0.72229^{6}^	0.72195^{5}^	0.62973^{4}^	0.62795^{3}^	0.76729^{9}^	0.14018^{1}^
		$\overset{ˆ}{\omega }$	0.00709^{2}^	0.00766^{6}^	0.00841^{7}^	0.00731^{3}^	0.00865^{8}^	0.00742^{4}^	0.00753^{5}^	0.00916^{10}^	0.00964^{11}^	0.00908^{9}^	0.01484^{12}^	0.00681^{1}^
	MRE	$\overset{ˆ}{\eta }$	0.35385^{1}^	0.3637^{5}^	0.37167^{8}^	0.36094^{3}^	0.37826^{10}^	0.37645^{9}^	0.36473^{6}^	0.36685^{7}^	0.35532^{2}^	0.36194^{4}^	0.39096^{11.5}^	0.39096^{11.5}^
		$\overset{ˆ}{\sigma }$	0.26121^{6}^	0.26404^{8}^	0.27944^{10}^	0.24319^{3}^	0.27384^{9}^	0.28668^{11}^	0.26302^{7}^	0.25881^{5}^	0.24257^{2}^	0.24378^{4}^	2.57074^{12}^	0.10792^{1}^
		$\overset{ˆ}{\omega }$	0.07537^{2}^	0.07818^{6}^	0.08188^{7}^	0.07658^{4}^	0.08281^{8}^	0.07632^{3}^	0.07726^{5}^	0.08549^{10}^	0.08768^{11}^	0.08511^{9}^	0.91184^{12}^	0.07176^{1}^
	∑*Ranks*		39^{2}^	77^{7}^	104^{10}^	40^{3}^	112^{11}^	97^{9}^	72^{6}^	90^{8}^	64^{4}^	71^{5}^	137^{12}^	33^{1}^
250	BIAS	$\overset{ˆ}{\eta }$	0.48135^{2}^	0.51665^{6}^	0.54702^{9}^	0.50164^{3}^	0.54776^{10}^	0.55193^{11}^	0.52075^{7}^	0.53091^{8}^	0.51451^{5}^	0.51052^{4}^	0.57335^{12}^	0.20963^{1}^
		$\overset{ˆ}{\sigma }$	0.5782^{4}^	0.6021^{6}^	0.68177^{11}^	0.53176^{2}^	0.66031^{10}^	0.70275^{12}^	0.60557^{7}^	0.61804^{9}^	0.58009^{5}^	0.5624^{3}^	0.61372^{8}^	0.22964^{1}^
		$\overset{ˆ}{\omega }$	0.05789^{4}^	0.0577^{2.5}^	0.06222^{8}^	0.0582^{5}^	0.06054^{7}^	0.0577^{2.5}^	0.05959^{6}^	0.06648^{10}^	0.06765^{11}^	0.06414^{9}^	0.08875^{12}^	0.05102^{1}^
	MSE	$\overset{ˆ}{\eta }$	0.34178^{2}^	0.38693^{5}^	0.42484^{9}^	0.37303^{3}^	0.42919^{10}^	0.43218^{11}^	0.39706^{7}^	0.40432^{8}^	0.3961^{6}^	0.38478^{4}^	0.46331^{12}^	0.08312^{1}^
		$\overset{ˆ}{\sigma }$	0.59599^{4}^	0.6272^{6}^	0.79083^{11}^	0.47931^{2}^	0.72322^{10}^	0.82561^{12}^	0.64203^{7}^	0.66116^{8}^	0.59661^{5}^	0.54681^{3}^	0.66839^{9}^	0.10357^{1}^
		$\overset{ˆ}{\omega }$	0.0051^{2}^	0.00522^{5}^	0.00595^{8}^	0.00514^{4}^	0.00563^{7}^	0.00511^{3}^	0.00547^{6}^	0.00683^{10}^	0.00704^{11}^	0.00634^{9}^	0.01184^{12}^	0.0042^{1}^
	MRE	$\overset{ˆ}{\eta }$	0.3209^{1}^	0.34443^{5}^	0.36468^{8}^	0.33443^{2}^	0.36518^{9}^	0.36795^{10}^	0.34717^{6}^	0.35394^{7}^	0.343^{4}^	0.34035^{3}^	0.38223^{11.5}^	0.38223^{11.5}^
		$\overset{ˆ}{\sigma }$	0.23128^{4}^	0.24084^{6}^	0.27271^{10}^	0.2127^{2}^	0.26412^{9}^	0.2811^{11}^	0.24223^{7}^	0.24722^{8}^	0.23204^{5}^	0.22496^{3}^	2.55223^{12}^	0.09186^{1}^
		$\overset{ˆ}{\omega }$	0.06433^{4}^	0.06411^{2.5}^	0.06914^{8}^	0.06467^{5}^	0.06726^{7}^	0.06411^{2.5}^	0.06622^{6}^	0.07387^{10}^	0.07516^{11}^	0.07126^{9}^	0.9052^{12}^	0.05669^{1}^
	∑*Ranks*		36^{2}^	57.5^{4}^	108^{11}^	37^{3}^	104^{10}^	98.5^{8}^	78^{6}^	103^{9}^	83^{7}^	62^{5}^	136^{12}^	33^{1}^

400	BIAS	$\overset{ˆ}{\eta }$	0.42945^{2}^	0.4791^{8}^	0.52136^{9}^	0.43629^{3}^	0.52819^{10}^	0.53195^{11}^	0.47714^{7}^	0.47642^{6}^	0.47399^{5}^	0.46301^{4}^	0.53929^{12}^	0.18649^{1}^
		$\overset{ˆ}{\sigma }$	0.49998^{4}^	0.53386^{8}^	0.61459^{10}^	0.44926^{2}^	0.62327^{11}^	0.65411^{12}^	0.52777^{6}^	0.52791^{7}^	0.5087^{5}^	0.48399^{3}^	0.55411^{9}^	0.20229^{1}^
		$\overset{ˆ}{\omega }$	0.04883^{3}^	0.05074^{6}^	0.05282^{7}^	0.04752^{2}^	0.05397^{9}^	0.04897^{4}^	0.05021^{5}^	0.05693^{11}^	0.05639^{10}^	0.05318^{8}^	0.07958^{12}^	0.04103^{1}^
	MSE	$\overset{ˆ}{\eta }$	0.27587^{2}^	0.34775^{8}^	0.39615^{9}^	0.29354^{3}^	0.40207^{10}^	0.40478^{11}^	0.34154^{6}^	0.33785^{5}^	0.34526^{7}^	0.3295^{4}^	0.41928^{12}^	0.06621^{1}^
		$\overset{ˆ}{\sigma }$	0.45665^{4}^	0.49842^{8}^	0.65201^{10}^	0.34101^{2}^	0.65489^{11}^	0.71749^{12}^	0.49332^{7}^	0.49179^{6}^	0.46292^{5}^	0.4086^{3}^	0.54527^{9}^	0.07935^{1}^
		$\overset{ˆ}{\omega }$	0.00371^{4}^	0.00393^{6}^	0.00428^{7}^	0.0035^{2}^	0.00447^{9}^	0.00368^{3}^	0.00387^{5}^	0.00493^{11}^	0.00491^{10}^	0.00435^{8}^	0.00938^{12}^	0.00268^{1}^
	MRE	$\overset{ˆ}{\eta }$	0.2863^{1}^	0.3194^{7}^	0.34757^{8}^	0.29086^{2}^	0.35213^{9}^	0.35463^{10}^	0.31809^{6}^	0.31761^{5}^	0.31599^{4}^	0.30867^{3}^	0.35953^{11.5}^	0.35953^{11.5}^
		$\overset{ˆ}{\sigma }$	0.19999^{4}^	0.21354^{8}^	0.24584^{9}^	0.17971^{2}^	0.24931^{10}^	0.26164^{11}^	0.21111^{6}^	0.21116^{7}^	0.20348^{5}^	0.1936^{3}^	2.54374^{12}^	0.08092^{1}^
		$\overset{ˆ}{\omega }$	0.05426^{3}^	0.05638^{6}^	0.05869^{7}^	0.0528^{2}^	0.05997^{9}^	0.05441^{4}^	0.05579^{5}^	0.06326^{11}^	0.06266^{10}^	0.05909^{8}^	0.90362^{12}^	0.04559^{1}^
	∑*Ranks*		35^{3}^	86^{7}^	100^{9}^	26^{1}^	116^{11}^	103^{10}^	70^{5}^	92^{8}^	80^{6}^	58^{4}^	137^{12}^	33^{2}^

500	BIAS	$\overset{ˆ}{\eta }$	0.39893^{2}^	0.44775^{5}^	0.50899^{10}^	0.41432^{3}^	0.50314^{9}^	0.52083^{11}^	0.44838^{6}^	0.46996^{8}^	0.4511^{7}^	0.43875^{4}^	0.53475^{12}^	0.17361^{1}^
		$\overset{ˆ}{\sigma }$	0.44831^{3}^	0.49563^{6}^	0.5906^{10}^	0.42893^{2}^	0.59912^{11}^	0.63589^{12}^	0.50652^{8}^	0.50305^{7}^	0.4829^{5}^	0.45866^{4}^	0.5381^{9}^	0.19023^{1}^
		$\overset{ˆ}{\omega }$	0.04443^{3}^	0.04656^{6}^	0.05116^{9}^	0.04434^{2}^	0.05098^{8}^	0.04618^{4}^	0.04654^{5}^	0.0539^{11}^	0.05138^{10}^	0.0491^{7}^	0.07631^{12}^	0.03705^{1}^
	MSE	$\overset{ˆ}{\eta }$	0.24539^{2}^	0.3061^{5}^	0.38006^{10}^	0.26686^{3}^	0.37116^{9}^	0.39345^{11}^	0.30673^{6}^	0.33508^{8}^	0.31536^{7}^	0.29691^{4}^	0.41442^{12}^	0.05693^{1}^
		$\overset{ˆ}{\sigma }$	0.36577^{3}^	0.43012^{6}^	0.60326^{10}^	0.32291^{2}^	0.62487^{11}^	0.68472^{12}^	0.46028^{8}^	0.44895^{7}^	0.41712^{5}^	0.37042^{4}^	0.52242^{9}^	0.07416^{1}^
		$\overset{ˆ}{\omega }$	0.00302^{2.5}^	0.00333^{6}^	0.00397^{9}^	0.00302^{2.5}^	0.00392^{8}^	0.00324^{4}^	0.0033^{5}^	0.00442^{11}^	0.0041^{10}^	0.00371^{7}^	0.00857^{12}^	0.00223^{1}^
	MRE	$\overset{ˆ}{\eta }$	0.26595^{1}^	0.2985^{4}^	0.33933^{9}^	0.27621^{2}^	0.33543^{8}^	0.34722^{10}^	0.29892^{5}^	0.31331^{7}^	0.30074^{6}^	0.2925^{3}^	0.3565^{11.5}^	0.3565^{11.5}^
		$\overset{ˆ}{\sigma }$	0.17932^{3}^	0.19825^{6}^	0.23624^{9}^	0.17157^{2}^	0.23965^{10}^	0.25436^{11}^	0.20261^{8}^	0.20122^{7}^	0.19316^{5}^	0.18346^{4}^	2.54075^{12}^	0.07609^{1}^
		$\overset{ˆ}{\omega }$	0.04937^{3}^	0.05173^{6}^	0.05684^{9}^	0.04926^{2}^	0.05664^{8}^	0.05131^{4}^	0.05172^{5}^	0.05989^{11}^	0.05709^{10}^	0.05455^{7}^	0.90237^{12}^	0.04117^{1}^
	∑*Ranks*		29.5^{2}^	66^{5}^	112^{11}^	26.5^{1}^	108^{10}^	104^{9}^	74^{6}^	102^{8}^	86^{7}^	58^{4}^	137^{12}^	33^{3}^

**Table 3:** Numerical values of simulation measures (BIAS, MSE, and MRE) for *η* = 0.5, *σ* = 2.0, *ω* = 1.5.

n	Est.	Est.Par.	*EM*_1_	*EM*_2_	*EM*_3_	*EM*_4_	*EM*_5_	*EM*_6_	*EM*_7_	*EM*_8_	*EM*_8_	*EM*_10_	*EM*_11_	*EM*_12_
20	BIAS	$\overset{ˆ}{\eta }$	0.19585^{2}^	0.20016^{5}^	0.20134^{6}^	0.20604^{11}^	0.20627^{12}^	0.1983^{4}^	0.20201^{8}^	0.20184^{7}^	0.19705^{3}^	0.20432^{9}^	0.2044^{10}^	0.16164^{1}^
		$\overset{ˆ}{\sigma }$	0.62636^{10}^	0.60818^{5}^	0.62645^{11}^	0.60379^{3}^	0.63552^{12}^	0.62216^{9}^	0.61857^{7}^	0.60576^{4}^	0.59699^{2}^	0.61918^{8}^	0.6185^{6}^	0.33912^{1}^
		$\overset{ˆ}{\omega }$	0.35336^{1}^	0.36492^{3}^	0.38485^{6}^	0.38058^{5}^	0.40144^{9}^	0.36105^{2}^	0.37713^{4}^	0.40105^{8}^	0.40442^{10}^	0.40928^{11}^	0.44882^{12}^	0.38705^{7}^
	MSE	$\overset{ˆ}{\eta }$	0.05308^{2}^	0.05553^{5}^	0.05602^{6}^	0.05789^{11}^	0.05814^{12}^	0.05479^{4}^	0.05631^{8}^	0.05616^{7}^	0.05441^{3}^	0.05695^{9}^	0.05699^{10}^	0.03977^{1}^
		$\overset{ˆ}{\sigma }$	0.64192^{12}^	0.5873^{5}^	0.61102^{10}^	0.55395^{2}^	0.61215^{11}^	0.60091^{8}^	0.58565^{4}^	0.59303^{7}^	0.55972^{3}^	0.59018^{6}^	0.61033^{9}^	0.21509^{1}^
		$\overset{ˆ}{\omega }$	0.20463^{2}^	0.21298^{5}^	0.23242^{6}^	0.20657^{3}^	0.23714^{7}^	0.20334^{1}^	0.21238^{4}^	0.24988^{11}^	0.24121^{8}^	0.24377^{9}^	0.29912^{12}^	0.24539^{10}^
	MRE	$\overset{ˆ}{\eta }$	0.3917^{1}^	0.40031^{4}^	0.40267^{5}^	0.41208^{11}^	0.41255^{12}^	0.3966^{3}^	0.40403^{7}^	0.40367^{6}^	0.3941^{2}^	0.40863^{8}^	0.40879^{9.5}^	0.40879^{9.5}^
		$\overset{ˆ}{\sigma }$	0.31318^{9}^	0.30409^{5}^	0.31322^{10}^	0.3019^{3}^	0.31776^{11}^	0.31108^{8}^	0.30928^{6}^	0.30288^{4}^	0.2985^{2}^	0.30959^{7}^	2.12386^{12}^	0.16956^{1}^
		$\overset{ˆ}{\omega }$	0.23557^{1}^	0.24328^{3}^	0.25656^{6}^	0.25372^{5}^	0.26762^{9}^	0.2407^{2}^	0.25142^{4}^	0.26736^{8}^	0.26961^{10}^	0.27285^{11}^	1.59321^{12}^	0.25803^{7}^
	∑*Ranks*		51^{1}^	52^{2}^	87^{9}^	73^{7}^	127^{12}^	54^{3}^	69^{6}^	80^{8}^	57^{5}^	104^{10}^	126^{11}^	56^{4}^

80	BIAS	$\overset{ˆ}{\eta }$	0.16948^{2}^	0.1814^{7}^	0.18777^{9}^	0.17156^{3}^	0.18835^{10}^	0.18995^{12}^	0.18418^{8}^	0.17988^{6}^	0.1761^{4}^	0.17964^{5}^	0.18912^{11}^	0.12427^{1}^
		$\overset{ˆ}{\sigma }$	0.54725^{8}^	0.54748^{9}^	0.56433^{11}^	0.48709^{3}^	0.55292^{10}^	0.56633^{12}^	0.513^{5}^	0.54427^{6}^	0.48647^{2}^	0.49939^{4}^	0.54488^{7}^	0.27492^{1}^
		$\overset{ˆ}{\omega }$	0.19992^{1}^	0.20427^{4}^	0.22186^{6}^	0.20312^{3}^	0.22243^{7}^	0.20044^{2}^	0.20887^{5}^	0.24407^{11}^	0.23871^{10}^	0.22769^{9}^	0.30818^{12}^	0.22518^{8}^
	MSE	$\overset{ˆ}{\eta }$	0.04103^{2}^	0.04648^{7}^	0.05014^{10}^	0.04315^{3}^	0.05075^{11}^	0.05135^{12}^	0.04842^{8}^	0.04602^{5}^	0.04498^{4}^	0.04612^{6}^	0.0494^{9}^	0.02517^{1}^
		$\overset{ˆ}{\sigma }$	0.52984^{12}^	0.50344^{7}^	0.52686^{10}^	0.41436^{3}^	0.50165^{6}^	0.52273^{9}^	0.43491^{5}^	0.51376^{8}^	0.40937^{2}^	0.4291^{4}^	0.52729^{11}^	0.14899^{1}^
		$\overset{ˆ}{\omega }$	0.06408^{3}^	0.06698^{4}^	0.07877^{8}^	0.06305^{2}^	0.07803^{6}^	0.06271^{1}^	0.06798^{5}^	0.09515^{11}^	0.08862^{10}^	0.07831^{7}^	0.1475^{12}^	0.08425^{9}^
	MRE	$\overset{ˆ}{\eta }$	0.33896^{1}^	0.36281^{6}^	0.37555^{8}^	0.34312^{2}^	0.3767^{9}^	0.37991^{12}^	0.36836^{7}^	0.35977^{5}^	0.3522^{3}^	0.35929^{4}^	0.37824^{10.5}^	0.37824^{10.5}^
		$\overset{ˆ}{\sigma }$	0.27362^{7}^	0.27374^{8}^	0.28216^{10}^	0.24355^{3}^	0.27646^{9}^	0.28317^{11}^	0.2565^{5}^	0.27214^{6}^	0.24324^{2}^	0.2497^{4}^	2.09002^{12}^	0.13746^{1}^
		$\overset{ˆ}{\omega }$	0.13328^{1}^	0.13618^{4}^	0.14791^{6}^	0.13542^{3}^	0.14829^{7}^	0.13363^{2}^	0.13924^{5}^	0.16271^{11}^	0.15914^{10}^	0.15179^{9}^	1.5505^{12}^	0.15012^{8}^
	∑*Ranks*		46^{2}^	74^{7}^	102^{11}^	33^{1}^	100^{10}^	98^{9}^	70^{6}^	91^{8}^	62^{4}^	69^{5}^	131^{12}^	60^{3}^

150	BIAS	$\overset{ˆ}{\eta }$	0.15346^{2}^	0.16447^{6}^	0.17816^{11}^	0.15372^{3}^	0.17815^{10}^	0.18073^{12}^	0.16816^{8}^	0.16483^{7}^	0.15928^{4}^	0.16003^{5}^	0.17394^{9}^	0.10209^{1}^
		$\overset{ˆ}{\sigma }$	0.50615^{8}^	0.48832^{7}^	0.51707^{10}^	0.43805^{3}^	0.51144^{9}^	0.54458^{12}^	0.47704^{5}^	0.48478^{6}^	0.42268^{2}^	0.45485^{4}^	0.52383^{11}^	0.23564^{1}^
		$\overset{ˆ}{\omega }$	0.149^{1}^	0.15529^{3}^	0.17476^{9}^	0.15784^{4}^	0.16919^{7}^	0.15355^{2}^	0.15793^{5}^	0.18373^{10}^	0.18745^{11}^	0.17363^{8}^	0.26137^{12}^	0.16043^{6}^
	MSE	$\overset{ˆ}{\eta }$	0.03402^{2}^	0.03937^{6}^	0.04598^{10}^	0.03514^{3}^	0.04611^{11}^	0.04697^{12}^	0.0416^{8}^	0.03947^{7}^	0.0382^{5}^	0.03743^{4}^	0.0421^{9}^	0.018^{1}^
		$\overset{ˆ}{\sigma }$	0.45441^{9}^	0.41976^{6}^	0.45768^{10}^	0.34697^{3}^	0.43898^{8}^	0.4924^{11}^	0.39735^{5}^	0.42021^{7}^	0.33236^{2}^	0.38154^{4}^	0.49569^{12}^	0.10844^{1}^
		$\overset{ˆ}{\omega }$	0.03472^{1}^	0.03814^{4}^	0.04807^{9}^	0.03783^{3}^	0.04508^{7}^	0.03728^{2}^	0.03894^{5}^	0.05361^{10}^	0.05504^{11}^	0.04665^{8}^	0.1046^{12}^	0.04213^{6}^
	MRE	$\overset{ˆ}{\eta }$	0.30691^{1}^	0.32894^{5}^	0.35631^{11}^	0.30743^{2}^	0.3563^{10}^	0.36146^{12}^	0.33632^{7}^	0.32967^{6}^	0.31857^{3}^	0.32006^{4}^	0.34789^{8.5}^	0.34789^{8.5}^
		$\overset{ˆ}{\sigma }$	0.25307^{8}^	0.24416^{7}^	0.25854^{10}^	0.21902^{3}^	0.25572^{9}^	0.27229^{11}^	0.23852^{5}^	0.24239^{6}^	0.21134^{2}^	0.22743^{4}^	2.05758^{12}^	0.11782^{1}^
		$\overset{ˆ}{\omega }$	0.09933^{1}^	0.10353^{3}^	0.11651^{9}^	0.10523^{4}^	0.11279^{7}^	0.10237^{2}^	0.10528^{5}^	0.12248^{10}^	0.12496^{11}^	0.11576^{8}^	1.51946^{12}^	0.10695^{6}^
	∑*Ranks*		43^{2}^	62^{4}^	119^{11}^	37^{1}^	104^{10}^	101^{9}^	70^{7}^	91^{8}^	67^{6}^	65^{5}^	130^{12}^	47^{3}^
250	BIAS	$\overset{ˆ}{\eta }$	0.13401^{3}^	0.14457^{6}^	0.16591^{11}^	0.12699^{2}^	0.16459^{10}^	0.17017^{12}^	0.15394^{8}^	0.14561^{7}^	0.13697^{4}^	0.13874^{5}^	0.1559^{9}^	0.08812^{1}^
		$\overset{ˆ}{\sigma }$	0.46008^{8}^	0.43497^{7}^	0.47264^{9}^	0.37724^{2}^	0.47283^{10}^	0.50651^{11}^	0.41735^{4}^	0.43414^{6}^	0.38151^{3}^	0.42167^{5}^	0.51561^{12}^	0.20849^{1}^
		$\overset{ˆ}{\omega }$	0.12088^{1}^	0.12768^{4}^	0.14286^{9}^	0.12411^{2}^	0.14271^{8}^	0.12738^{3}^	0.12995^{6}^	0.15178^{11}^	0.149^{10}^	0.14249^{7}^	0.22071^{12}^	0.12817^{5}^
	MSE	$\overset{ˆ}{\eta }$	0.0261^{3}^	0.03121^{6}^	0.04112^{11}^	0.02461^{2}^	0.04035^{10}^	0.04212^{12}^	0.03601^{9}^	0.03164^{7}^	0.02874^{5}^	0.02851^{4}^	0.03404^{8}^	0.01387^{1}^
		$\overset{ˆ}{\sigma }$	0.38232^{8}^	0.34044^{6}^	0.39098^{10}^	0.26827^{2}^	0.39077^{9}^	0.4327^{11}^	0.30715^{4}^	0.34401^{7}^	0.28469^{3}^	0.3398^{5}^	0.47279^{12}^	0.0864^{1}^
		$\overset{ˆ}{\omega }$	0.0225^{1}^	0.02536^{4}^	0.03166^{8}^	0.02363^{2}^	0.03204^{9}^	0.02531^{3}^	0.02656^{5}^	0.03648^{11}^	0.03437^{10}^	0.0312^{7}^	0.07358^{12}^	0.02684^{6}^
	MRE	$\overset{ˆ}{\eta }$	0.26802^{2}^	0.28913^{5}^	0.33182^{11}^	0.25398^{1}^	0.32918^{10}^	0.34035^{12}^	0.30788^{7}^	0.29122^{6}^	0.27394^{3}^	0.27747^{4}^	0.3118^{8.5}^	0.3118^{8.5}^
		$\overset{ˆ}{\sigma }$	0.23004^{8}^	0.21749^{7}^	0.23632^{9}^	0.18862^{2}^	0.23642^{10}^	0.25325^{11}^	0.20868^{4}^	0.21707^{6}^	0.19076^{3}^	0.21084^{5}^	2.04715^{12}^	0.10424^{1}^
		$\overset{ˆ}{\omega }$	0.08059^{1}^	0.08512^{4}^	0.09524^{9}^	0.08274^{2}^	0.09514^{8}^	0.08492^{3}^	0.08663^{6}^	0.10119^{11}^	0.09933^{10}^	0.095^{7}^	1.51547^{12}^	0.08545^{5}^
	∑*Ranks*		46^{3}^	65^{4.5}^	116^{11}^	22^{1}^	112^{10}^	104^{9}^	70^{7}^	95^{8}^	67^{6}^	65^{4.5}^	130^{12}^	44^{2}^

400	BIAS	$\overset{ˆ}{\eta }$	0.11463^{3}^	0.12685^{5}^	0.14832^{9}^	0.10781^{2}^	0.15023^{11}^	0.15676^{12}^	0.12749^{7}^	0.1305^{8}^	0.12154^{4}^	0.12743^{6}^	0.14834^{10}^	0.07528^{1}^
		$\overset{ˆ}{\sigma }$	0.4042^{7}^	0.37855^{5}^	0.41918^{9}^	0.33778^{2}^	0.41829^{8}^	0.45988^{11}^	0.34844^{3}^	0.38819^{6}^	0.37218^{4}^	0.42334^{10}^	0.52532^{12}^	0.18663^{1}^
		$\overset{ˆ}{\omega }$	0.09838^{1}^	0.105^{4}^	0.11797^{8}^	0.1007^{2}^	0.12022^{9}^	0.10697^{6}^	0.10556^{5}^	0.12726^{11}^	0.12332^{10}^	0.1162^{7}^	0.19951^{12}^	0.10285^{3}^
	MSE	$\overset{ˆ}{\eta }$	0.01909^{3}^	0.02455^{6}^	0.03352^{10}^	0.01763^{2}^	0.0342^{11}^	0.03707^{12}^	0.0258^{8}^	0.02534^{7}^	0.02215^{4}^	0.02382^{5}^	0.03029^{9}^	0.01049^{1}^
		$\overset{ˆ}{\sigma }$	0.29209^{7}^	0.2631^{5}^	0.31291^{9}^	0.21628^{2}^	0.30777^{8}^	0.36847^{11}^	0.21967^{3}^	0.27845^{6}^	0.2589^{4}^	0.32181^{10}^	0.47803^{12}^	0.07191^{1}^
		$\overset{ˆ}{\omega }$	0.015^{1}^	0.0176^{5}^	0.02188^{8}^	0.01567^{2}^	0.02225^{9}^	0.01792^{6}^	0.01745^{4}^	0.02522^{11}^	0.0236^{10}^	0.02086^{7}^	0.05875^{12}^	0.01703^{3}^
	MRE	$\overset{ˆ}{\eta }$	0.22927^{2}^	0.25371^{4}^	0.29664^{8}^	0.21562^{1}^	0.30047^{11}^	0.31353^{12}^	0.25499^{6}^	0.26099^{7}^	0.24308^{3}^	0.25486^{5}^	0.29667^{9.5}^	0.29667^{9.5}^
		$\overset{ˆ}{\sigma }$	0.2021^{7}^	0.18927^{5}^	0.20959^{9}^	0.16889^{2}^	0.20915^{8}^	0.22994^{11}^	0.17422^{3}^	0.1941^{6}^	0.18609^{4}^	0.21167^{10}^	2.04847^{12}^	0.09332^{1}^
		$\overset{ˆ}{\omega }$	0.06558^{1}^	0.07^{4}^	0.07865^{8}^	0.06714^{2}^	0.08015^{9}^	0.07131^{6}^	0.07037^{5}^	0.08484^{11}^	0.08221^{10}^	0.07746^{7}^	1.51102^{12}^	0.06856^{3}^
	∑*Ranks*		42^{3}^	56^{4}^	103^{9}^	22^{1}^	112^{10}^	116^{11}^	58^{5}^	97^{8}^	70^{6}^	89^{7}^	134^{12}^	37^{2}^

500	BIAS	$\overset{ˆ}{\eta }$	0.10754^{3}^	0.11481^{4}^	0.14418^{10}^	0.10169^{2}^	0.1429^{9}^	0.1491^{12}^	0.12006^{8}^	0.11836^{6}^	0.11825^{5}^	0.11994^{7}^	0.14576^{11}^	0.07051^{1}^
		$\overset{ˆ}{\sigma }$	0.37719^{7}^	0.34052^{4}^	0.40345^{10}^	0.32149^{3}^	0.38932^{8}^	0.4256^{11}^	0.31531^{2}^	0.35432^{5}^	0.37509^{6}^	0.40256^{9}^	0.51675^{12}^	0.17778^{1}^
		$\overset{ˆ}{\omega }$	0.08913^{1}^	0.0953^{4}^	0.10996^{8}^	0.09193^{2}^	0.11142^{9}^	0.09944^{6}^	0.09577^{5}^	0.11476^{11}^	0.11315^{10}^	0.10506^{7}^	0.18878^{12}^	0.09207^{3}^
	MSE	$\overset{ˆ}{\eta }$	0.01695^{3}^	0.02041^{4}^	0.03183^{11}^	0.01538^{2}^	0.03163^{10}^	0.03393^{12}^	0.02324^{8}^	0.02135^{7}^	0.02087^{6}^	0.02084^{5}^	0.02875^{9}^	0.00893^{1}^
		$\overset{ˆ}{\sigma }$	0.26112^{7}^	0.21207^{4}^	0.29279^{10}^	0.19141^{3}^	0.26353^{8}^	0.31669^{11}^	0.17727^{2}^	0.23807^{5}^	0.2513^{6}^	0.2719^{9}^	0.44726^{12}^	0.06206^{1}^
		$\overset{ˆ}{\omega }$	0.01241^{1}^	0.0141^{4}^	0.01897^{8}^	0.01292^{2}^	0.01951^{9}^	0.01539^{6}^	0.01453^{5}^	0.02063^{11}^	0.01972^{10}^	0.017^{7}^	0.05253^{12}^	0.01381^{3}^
	MRE	$\overset{ˆ}{\eta }$	0.21509^{2}^	0.22963^{3}^	0.28836^{9}^	0.20339^{1}^	0.2858^{8}^	0.29821^{12}^	0.24011^{7}^	0.23672^{5}^	0.2365^{4}^	0.23987^{6}^	0.29153^{10.5}^	0.29153^{10.5}^
		$\overset{ˆ}{\sigma }$	0.18859^{7}^	0.17026^{4}^	0.20172^{10}^	0.16075^{3}^	0.19466^{8}^	0.2128^{11}^	0.15766^{2}^	0.17716^{5}^	0.18754^{6}^	0.20128^{9}^	2.04592^{12}^	0.08889^{1}^
		$\overset{ˆ}{\omega }$	0.05942^{1}^	0.06354^{4}^	0.07331^{8}^	0.06129^{2}^	0.07428^{9}^	0.0663^{6}^	0.06385^{5}^	0.07651^{11}^	0.07543^{10}^	0.07004^{7}^	1.50439^{12}^	0.06138^{3}^
	∑*Ranks*		42^{3}^	46^{4}^	111^{10}^	26^{1}^	103^{9}^	116^{11}^	58^{5}^	87^{7}^	83^{6}^	88^{8}^	137^{12}^	39^{2}^

**Table 4:** Numerical values of simulation measures (BIAS, MSE, and MRE) for *η* = 0.9, *σ* = 4.0, *ω* = 2.5.

n	Est.	Est.Par.	*EM*_1_	*EM*_2_	*EM*_3_	*EM*_4_	*EM*_5_	*EM*_6_	*EM*_7_	*EM*_8_	*EM*_8_	*EM*_10_	*EM*_11_	*EM*_12_
20	BIAS	$\overset{ˆ}{\eta }$	0.36267^{5}^	0.35902^{4}^	0.3689^{6}^	0.37065^{9}^	0.36973^{7}^	0.37537^{11}^	0.37059^{8}^	0.37246^{10}^	0.35728^{3}^	0.35558^{2}^	0.37906^{12}^	0.28191^{1}^
		$\overset{ˆ}{\sigma }$	1.31074^{6}^	1.30224^{5}^	1.35443^{10}^	1.33807^{8}^	1.40568^{12}^	1.36012^{11}^	1.34032^{9}^	1.29985^{4}^	1.17196^{2}^	1.23959^{3}^	1.32664^{7}^	0.56673^{1}^
		$\overset{ˆ}{\omega }$	0.42268^{1}^	0.42577^{2}^	0.46379^{5}^	0.48419^{8}^	0.49082^{9}^	0.44371^{3}^	0.466^{6}^	0.46374^{4}^	0.51876^{11}^	0.52021^{12}^	0.51183^{10}^	0.48361^{7}^
	MSE	$\overset{ˆ}{\eta }$	0.18356^{5}^	0.18054^{4}^	0.18966^{8}^	0.19042^{9}^	0.18869^{6}^	0.19348^{11}^	0.18876^{7}^	0.19263^{10}^	0.17426^{3}^	0.17247^{2}^	0.19783^{12}^	0.12288^{1}^
		$\overset{ˆ}{\sigma }$	2.62413^{8}^	2.54219^{5}^	2.76331^{11}^	2.5819^{7}^	2.84449^{12}^	2.75266^{10}^	2.57142^{6}^	2.54215^{4}^	2.26998^{2}^	2.47661^{3}^	2.64467^{9}^	0.67831^{1}^
		$\overset{ˆ}{\omega }$	0.29356^{2}^	0.2846^{1}^	0.33871^{5}^	0.33887^{6}^	0.35727^{8}^	0.30204^{3}^	0.32112^{4}^	0.34003^{7}^	0.40335^{11}^	0.39468^{10}^	0.41838^{12}^	0.3936^{9}^
	MRE	$\overset{ˆ}{\eta }$	0.40297^{4}^	0.39891^{3}^	0.40989^{5}^	0.41184^{8}^	0.41081^{6}^	0.41708^{10}^	0.41177^{7}^	0.41385^{9}^	0.39698^{2}^	0.39509^{1}^	0.42117^{11.5}^	0.42117^{11.5}^
		$\overset{ˆ}{\sigma }$	0.32768^{6}^	0.32556^{5}^	0.33861^{9}^	0.33452^{7}^	0.35142^{11}^	0.34003^{10}^	0.33508^{8}^	0.32496^{4}^	0.29299^{2}^	0.3099^{3}^	4.29071^{12}^	0.14168^{1}^
		$\overset{ˆ}{\omega }$	0.16907^{1}^	0.17031^{2}^	0.18552^{5}^	0.19368^{8}^	0.19633^{9}^	0.17748^{3}^	0.1864^{6}^	0.1855^{4}^	0.2075^{10}^	0.20808^{11}^	2.6035^{12}^	0.19344^{7}^
	∑*Ranks*		49^{2}^	41^{1}^	83^{8}^	93^{9}^	106^{11}^	95^{10}^	82^{7}^	73^{6}^	60^{4}^	62^{5}^	133^{12}^	59^{3}^

80	BIAS	$\overset{ˆ}{\eta }$	0.3393^{4}^	0.34811^{7}^	0.35034^{9}^	0.34625^{6}^	0.35076^{10}^	0.36104^{12}^	0.34508^{5}^	0.34851^{8}^	0.3226^{2}^	0.33756^{3}^	0.3544^{11}^	0.18426^{1}^
		$\overset{ˆ}{\sigma }$	1.16834^{6}^	1.18743^{8}^	1.23057^{11}^	1.13815^{3}^	1.22114^{10}^	1.23916^{12}^	1.1941^{9}^	1.15109^{4}^	1.10749^{2}^	1.15133^{5}^	1.17904^{7}^	0.39485^{1}^
		$\overset{ˆ}{\omega }$	0.22755^{1}^	0.23613^{3}^	0.25383^{7}^	0.23757^{4}^	0.25353^{6}^	0.23924^{5}^	0.23551^{2}^	0.25647^{8}^	0.29095^{11}^	0.26766^{10}^	0.30294^{12}^	0.26133^{9}^
	MSE	$\overset{ˆ}{\eta }$	0.16374^{3}^	0.1706^{6}^	0.1732^{10}^	0.17233^{7}^	0.17266^{9}^	0.18339^{12}^	0.16818^{5}^	0.17258^{8}^	0.15091^{2}^	0.16407^{4}^	0.17596^{11}^	0.05874^{1}^
		$\overset{ˆ}{\sigma }$	2.21247^{6}^	2.26234^{9}^	2.41575^{12}^	2.00448^{2}^	2.31871^{10}^	2.38684^{11}^	2.2271^{7}^	2.11108^{4}^	2.0384^{3}^	2.15049^{5}^	2.23456^{8}^	0.32201^{1}^
		$\overset{ˆ}{\omega }$	0.08206^{1}^	0.0879^{4}^	0.10204^{7}^	0.08689^{3}^	0.10083^{6}^	0.08984^{5}^	0.08589^{2}^	0.10456^{8}^	0.13178^{11}^	0.11159^{10}^	0.14641^{12}^	0.11111^{9}^
	MRE	$\overset{ˆ}{\eta }$	0.377^{3}^	0.38679^{6}^	0.38927^{8}^	0.38473^{5}^	0.38973^{9}^	0.40116^{12}^	0.38342^{4}^	0.38723^{7}^	0.35844^{1}^	0.37507^{2}^	0.39378^{10.5}^	0.39378^{10.5}^
		$\overset{ˆ}{\sigma }$	0.29208^{6}^	0.29686^{7}^	0.30764^{10}^	0.28454^{3}^	0.30529^{9}^	0.30979^{11}^	0.29852^{8}^	0.28777^{4}^	0.27687^{2}^	0.28783^{5}^	4.14354^{12}^	0.09871^{1}^
		$\overset{ˆ}{\omega }$	0.09102^{1}^	0.09445^{3}^	0.10153^{7}^	0.09503^{4}^	0.10141^{6}^	0.09569^{5}^	0.0942^{2}^	0.10259^{8}^	0.11638^{11}^	0.10706^{10}^	2.54789^{12}^	0.10453^{9}^
	∑*Ranks*		41^{1}^	69^{6}^	106^{10}^	49^{2}^	99^{9}^	113^{11}^	58^{3}^	78^{8}^	59^{4}^	71^{7}^	130^{12}^	63^{5}^

150	BIAS	$\overset{ˆ}{\eta }$	0.32469^{3}^	0.33268^{4}^	0.34421^{12}^	0.33313^{5}^	0.34286^{10}^	0.33827^{9}^	0.33545^{8}^	0.33533^{7}^	0.30778^{2}^	0.33491^{6}^	0.34345^{11}^	0.14463^{1}^
		$\overset{ˆ}{\sigma }$	1.12204^{7}^	1.15271^{9}^	1.18857^{12}^	1.0756^{3}^	1.18833^{11}^	1.18742^{10}^	1.11343^{6}^	1.12669^{8}^	1.03125^{2}^	1.09804^{4}^	1.10873^{5}^	0.34804^{1}^
		$\overset{ˆ}{\omega }$	0.17951^{1}^	0.18103^{2}^	0.19312^{7}^	0.18181^{3}^	0.19503^{8}^	0.18216^{4}^	0.18371^{5}^	0.20427^{10}^	0.21826^{11}^	0.20099^{9}^	0.24119^{12}^	0.19271^{6}^
	MSE	$\overset{ˆ}{\eta }$	0.15192^{3}^	0.15837^{4}^	0.16811^{12}^	0.15921^{5}^	0.16725^{11}^	0.16381^{9}^	0.16142^{8}^	0.1607^{6}^	0.14116^{2}^	0.16076^{7}^	0.16711^{10}^	0.03725^{1}^
		$\overset{ˆ}{\sigma }$	2.07675^{8}^	2.1495^{9}^	2.26106^{12}^	1.83568^{3}^	2.21881^{10}^	2.24053^{11}^	1.97141^{5}^	2.0618^{7}^	1.8269^{2}^	1.95247^{4}^	2.03437^{6}^	0.24658^{1}^
		$\overset{ˆ}{\omega }$	0.05025^{1}^	0.05177^{4}^	0.05817^{6}^	0.05061^{2}^	0.05953^{7}^	0.05158^{3}^	0.05274^{5}^	0.06449^{10}^	0.07472^{11}^	0.06269^{9}^	0.09028^{12}^	0.06003^{8}^
	MRE	$\overset{ˆ}{\eta }$	0.36077^{2}^	0.36965^{3}^	0.38246^{12}^	0.37014^{4}^	0.38095^{9}^	0.37585^{8}^	0.37272^{7}^	0.37259^{6}^	0.34198^{1}^	0.37212^{5}^	0.38161^{10.5}^	0.38161^{10.5}^
		$\overset{ˆ}{\sigma }$	0.28051^{6}^	0.28818^{8}^	0.29714^{11}^	0.2689^{3}^	0.29708^{10}^	0.29686^{9}^	0.27836^{5}^	0.28167^{7}^	0.25781^{2}^	0.27451^{4}^	4.11613^{12}^	0.08701^{1}^
		$\overset{ˆ}{\omega }$	0.07181^{1}^	0.07241^{2}^	0.07725^{7}^	0.07273^{3}^	0.07801^{8}^	0.07286^{4}^	0.07348^{5}^	0.08171^{10}^	0.0873^{11}^	0.0804^{9}^	2.52625^{12}^	0.07708^{6}^
	∑*Ranks*		41^{1.5}^	58^{4.5}^	121^{11}^	41^{1.5}^	111^{10}^	88^{8}^	71^{6}^	94^{9}^	58^{4.5}^	75^{7}^	125^{12}^	53^{3}^
250	BIAS	$\overset{ˆ}{\eta }$	0.30512^{3}^	0.3235^{6}^	0.33824^{10}^	0.32029^{4}^	0.33932^{11}^	0.33466^{9}^	0.32813^{8}^	0.32623^{7}^	0.30263^{2}^	0.32149^{5}^	0.33965^{12}^	0.11581^{1}^
		$\overset{ˆ}{\sigma }$	1.0703^{6}^	1.10325^{9}^	1.14641^{10}^	0.99826^{3}^	1.16603^{11}^	1.16926^{12}^	1.10275^{8}^	1.07942^{7}^	0.99745^{2}^	1.04417^{4}^	1.06391^{5}^	0.30237^{1}^
		$\overset{ˆ}{\omega }$	0.14665^{2}^	0.15389^{6}^	0.16119^{9}^	0.14716^{3}^	0.16091^{8}^	0.14877^{4}^	0.15035^{5}^	0.16558^{10}^	0.1763^{11}^	0.16066^{7}^	0.20902^{12}^	0.14642^{1}^
	MSE	$\overset{ˆ}{\eta }$	0.13622^{2}^	0.15058^{4}^	0.16322^{11}^	0.15162^{6}^	0.16198^{10}^	0.16164^{9}^	0.15529^{8}^	0.15407^{7}^	0.13761^{3}^	0.15123^{5}^	0.1648^{12}^	0.02428^{1}^
		$\overset{ˆ}{\sigma }$	1.91667^{7}^	2.00135^{9}^	2.11538^{10}^	1.61567^{2}^	2.16009^{11}^	2.16327^{12}^	1.97162^{8}^	1.91072^{6}^	1.71293^{3}^	1.78466^{4}^	1.91004^{5}^	0.18964^{1}^
		$\overset{ˆ}{\omega }$	0.03361^{2}^	0.03675^{6}^	0.04041^{8}^	0.0335^{1}^	0.0398^{7}^	0.03466^{4}^	0.03569^{5}^	0.04269^{10}^	0.04859^{11}^	0.04045^{9}^	0.06711^{12}^	0.03403^{3}^
	MRE	$\overset{ˆ}{\eta }$	0.33902^{2}^	0.35944^{5}^	0.37582^{9}^	0.35588^{3}^	0.37702^{10}^	0.37184^{8}^	0.36459^{7}^	0.36248^{6}^	0.33625^{1}^	0.35721^{4}^	0.37739^{11.5}^	0.37739^{11.5}^
		$\overset{ˆ}{\sigma }$	0.26758^{5}^	0.27581^{8}^	0.2866^{9}^	0.24957^{3}^	0.29151^{10}^	0.29232^{11}^	0.27569^{7}^	0.26986^{6}^	0.24936^{2}^	0.26104^{4}^	4.09787^{12}^	0.07559^{1}^
		$\overset{ˆ}{\omega }$	0.05866^{2}^	0.06156^{6}^	0.06448^{9}^	0.05886^{3}^	0.06436^{8}^	0.05951^{4}^	0.06014^{5}^	0.06623^{10}^	0.07052^{11}^	0.06426^{7}^	2.51064^{12}^	0.05857^{1}^
	∑*Ranks*		40^{3}^	78^{6}^	112^{10}^	37^{2}^	114^{11}^	96^{9}^	80^{7}^	91^{8}^	60^{4}^	64^{5}^	129^{12}^	35^{1}^

400	BIAS	$\overset{ˆ}{\eta }$	0.28195^{2}^	0.30523^{5}^	0.3238^{9}^	0.29563^{4}^	0.32419^{10}^	0.32915^{11}^	0.30823^{7}^	0.30881^{8}^	0.2935^{3}^	0.3059^{6}^	0.33232^{12}^	0.10105^{1}^
		$\overset{ˆ}{\sigma }$	0.97488^{5}^	1.04143^{9}^	1.11067^{11}^	0.91603^{2}^	1.1021^{10}^	1.13956^{12}^	1.02503^{8}^	1.00838^{6}^	0.94427^{3}^	0.96472^{4}^	1.01861^{7}^	0.27415^{1}^
		$\overset{ˆ}{\omega }$	0.12456^{2}^	0.12802^{6}^	0.13531^{8}^	0.12496^{3}^	0.13386^{7}^	0.12579^{4}^	0.12643^{5}^	0.14581^{10}^	0.14694^{11}^	0.13684^{9}^	0.1891^{12}^	0.12327^{1}^
	MSE	$\overset{ˆ}{\eta }$	0.11813^{2}^	0.13723^{5}^	0.15173^{9}^	0.13143^{3}^	0.15267^{10}^	0.15751^{11}^	0.14113^{8}^	0.14064^{7}^	0.13174^{4}^	0.13971^{6}^	0.15856^{12}^	0.01869^{1}^
		$\overset{ˆ}{\sigma }$	1.64524^{5}^	1.80979^{9}^	1.98888^{11}^	1.38297^{2}^	1.97578^{10}^	2.07999^{12}^	1.76011^{8}^	1.72556^{6}^	1.55828^{4}^	1.5561^{3}^	1.74441^{7}^	0.15711^{1}^
		$\overset{ˆ}{\omega }$	0.02429^{3}^	0.02517^{6}^	0.02877^{8}^	0.02403^{2}^	0.02778^{7}^	0.0247^{4}^	0.025^{5}^	0.03251^{10}^	0.03324^{11}^	0.02886^{9}^	0.05381^{12}^	0.02394^{1}^
	MRE	$\overset{ˆ}{\eta }$	0.31328^{1}^	0.33914^{4}^	0.35977^{8}^	0.32847^{3}^	0.36021^{9}^	0.36573^{10}^	0.34248^{6}^	0.34312^{7}^	0.32611^{2}^	0.33988^{5}^	0.36924^{11.5}^	0.36924^{11.5}^
		$\overset{ˆ}{\sigma }$	0.24372^{5}^	0.26036^{8}^	0.27767^{10}^	0.22901^{2}^	0.27552^{9}^	0.28489^{11}^	0.25626^{7}^	0.2521^{6}^	0.23607^{3}^	0.24118^{4}^	4.09468^{12}^	0.06854^{1}^
		$\overset{ˆ}{\omega }$	0.04982^{2}^	0.05121^{6}^	0.05412^{8}^	0.04998^{3}^	0.05355^{7}^	0.05032^{4}^	0.05057^{5}^	0.05832^{10}^	0.05878^{11}^	0.05473^{9}^	2.51047^{12}^	0.04931^{1}^
	∑*Ranks*		35^{3}^	76^{6}^	108^{11}^	32^{1}^	104^{9.5}^	104^{9.5}^	77^{7}^	93^{8}^	68^{4}^	73^{5}^	133^{12}^	33^{2}^

500	BIAS	$\overset{ˆ}{\eta }$	0.27552^{2}^	0.2964^{6}^	0.31881^{9}^	0.28732^{4}^	0.31912^{10}^	0.3239^{11}^	0.29094^{5}^	0.30124^{8}^	0.28361^{3}^	0.29654^{7}^	0.33189^{12}^	0.09389^{1}^
		$\overset{ˆ}{\sigma }$	0.93391^{5}^	1.00839^{8}^	1.10463^{11}^	0.87462^{2}^	1.09188^{10}^	1.12225^{12}^	0.98824^{6}^	0.99084^{7}^	0.91175^{3}^	0.92507^{4}^	1.0142^{9}^	0.26098^{1}^
		$\overset{ˆ}{\omega }$	0.11513^{4}^	0.11811^{6}^	0.1249^{7}^	0.11452^{2}^	0.12626^{8}^	0.11489^{3}^	0.11739^{5}^	0.13616^{11}^	0.13339^{10}^	0.12813^{9}^	0.18421^{12}^	0.10947^{1}^
	MSE	$\overset{ˆ}{\eta }$	0.11323^{2}^	0.13126^{6}^	0.14856^{9}^	0.12688^{5}^	0.14943^{10}^	0.15247^{11}^	0.126^{4}^	0.13469^{8}^	0.12452^{3}^	0.13233^{7}^	0.15759^{12}^	0.01619^{1}^
		$\overset{ˆ}{\sigma }$	1.49779^{5}^	1.7267^{8}^	2.00061^{11}^	1.28761^{2}^	1.96531^{10}^	2.02196^{12}^	1.66141^{6}^	1.6678^{7}^	1.44293^{4}^	1.41885^{3}^	1.73424^{9}^	0.14411^{1}^
		$\overset{ˆ}{\omega }$	0.02052^{3}^	0.02159^{6}^	0.02407^{7}^	0.01999^{2}^	0.02452^{8}^	0.02077^{4}^	0.02141^{5}^	0.02851^{11}^	0.02742^{10}^	0.02539^{9}^	0.05032^{12}^	0.01906^{1}^
	MRE	$\overset{ˆ}{\eta }$	0.30613^{1}^	0.32933^{5}^	0.35423^{8}^	0.31924^{3}^	0.35458^{9}^	0.35989^{10}^	0.32327^{4}^	0.33471^{7}^	0.31513^{2}^	0.32949^{6}^	0.36876^{11.5}^	0.36876^{11.5}^
		$\overset{ˆ}{\sigma }$	0.23348^{5}^	0.2521^{8}^	0.27616^{10}^	0.21866^{2}^	0.27297^{9}^	0.28056^{11}^	0.24706^{6}^	0.24771^{7}^	0.22794^{3}^	0.23127^{4}^	4.07806^{12}^	0.06524^{1}^
		$\overset{ˆ}{\omega }$	0.04605^{4}^	0.04724^{6}^	0.04996^{7}^	0.04581^{2}^	0.05051^{8}^	0.04596^{3}^	0.04696^{5}^	0.05446^{11}^	0.05336^{10}^	0.05125^{9}^	2.50518^{12}^	0.04379^{1}^
	∑*Ranks*		41^{3}^	78^{7}^	104^{10}^	31^{1}^	108^{11}^	101^{8}^	61^{4}^	102^{9}^	63^{5}^	77^{6}^	137^{12}^	33^{2}^

**Table 5:** Numerical values of simulation measures (BIAS, MSE, and MRE) for *η* = 2.0, *σ* = 3.0, *ω* = 2.0.

n	Est.	Est.Par.	*EM*_1_	*EM*_2_	*EM*_3_	*EM*_4_	*EM*_5_	*EM*_6_	*EM*_7_	*EM*_8_	*EM*_8_	*EM*_10_	*EM*_11_	*EM*_12_
20	BIAS	$\overset{ˆ}{\eta }$	0.82111^{4}^	0.83554^{5}^	0.84643^{8}^	0.8508^{9}^	0.86929^{11}^	0.83869^{6}^	0.86015^{10}^	0.84249^{7}^	0.78733^{2}^	0.78999^{3}^	0.87615^{12}^	0.55664^{1}^
		$\overset{ˆ}{\sigma }$	0.96237^{2}^	0.96944^{4}^	1.02334^{11}^	0.98912^{7}^	1.03059^{12}^	1.01157^{9}^	0.96727^{3}^	0.97982^{6}^	0.97401^{5}^	1.01225^{10}^	0.99949^{8}^	0.61902^{1}^
		$\overset{ˆ}{\omega }$	0.30495^{1}^	0.31828^{2}^	0.33717^{5}^	0.36491^{10}^	0.37143^{12}^	0.32363^{3}^	0.34073^{6}^	0.34466^{7}^	0.36178^{8}^	0.36336^{9}^	0.37099^{11}^	0.33354^{4}^
	MSE	$\overset{ˆ}{\eta }$	0.93255^{4}^	0.94912^{5}^	0.98527^{9}^	0.98031^{8}^	1.02127^{11}^	0.97227^{7}^	0.99937^{10}^	0.96725^{6}^	0.86845^{3}^	0.86702^{2}^	1.03168^{12}^	0.5099^{1}^
		$\overset{ˆ}{\sigma }$	1.45438^{6}^	1.43745^{5}^	1.57049^{12}^	1.41759^{3}^	1.55937^{11}^	1.54754^{10}^	1.37765^{2}^	1.46861^{7}^	1.42735^{4}^	1.50616^{8}^	1.54196^{9}^	0.71782^{1}^
		$\overset{ˆ}{\omega }$	0.1489^{1}^	0.15643^{2}^	0.17675^{5}^	0.19189^{8}^	0.20269^{11}^	0.16214^{3}^	0.17206^{4}^	0.18468^{6}^	0.20104^{10}^	0.19693^{9}^	0.2127^{12}^	0.18972^{7}^
	MRE	$\overset{ˆ}{\eta }$	0.41055^{3}^	0.41777^{4}^	0.42322^{7}^	0.4254^{8}^	0.43465^{10}^	0.41935^{5}^	0.43008^{9}^	0.42124^{6}^	0.39367^{1}^	0.39499^{2}^	0.43807^{11.5}^	0.43807^{11.5}^
		$\overset{ˆ}{\sigma }$	0.32079^{2}^	0.32315^{4}^	0.34111^{10}^	0.32971^{7}^	0.34353^{11}^	0.33719^{8}^	0.32242^{3}^	0.32661^{6}^	0.32467^{5}^	0.33742^{9}^	3.24518^{12}^	0.20634^{1}^
		$\overset{ˆ}{\omega }$	0.15247^{1}^	0.15914^{2}^	0.16859^{5}^	0.18246^{10}^	0.18572^{11}^	0.16182^{3}^	0.17037^{6}^	0.17233^{7}^	0.18089^{8}^	0.18168^{9}^	2.07669^{12}^	0.16677^{4}^
	∑*Ranks*		30^{1}^	43^{2}^	94^{9}^	95^{10}^	132^{11}^	70^{5}^	71^{6}^	77^{7}^	60^{4}^	81^{8}^	135^{12}^	48^{3}^

80	BIAS	$\overset{ˆ}{\eta }$	0.76088^{3}^	0.77344^{5}^	0.78468^{8}^	0.7895^{10}^	0.7913^{11}^	0.78596^{9}^	0.77935^{6}^	0.78005^{7}^	0.73601^{2}^	0.76987^{4}^	0.81113^{12}^	0.37809^{1}^
		$\overset{ˆ}{\sigma }$	0.88092^{9}^	0.87403^{7}^	0.90024^{10}^	0.84224^{3}^	0.90148^{12}^	0.90032^{11}^	0.87793^{8}^	0.85238^{5}^	0.8317^{2}^	0.84785^{4}^	0.86467^{6}^	0.41225^{1}^
		$\overset{ˆ}{\omega }$	0.17368^{2}^	0.18011^{3}^	0.1912^{7}^	0.18471^{5}^	0.19179^{8}^	0.18702^{6}^	0.18026^{4}^	0.1944^{9}^	0.20642^{11}^	0.20045^{10}^	0.2259^{12}^	0.17198^{1}^
	MSE	$\overset{ˆ}{\eta }$	0.81559^{3}^	0.8352^{4}^	0.86465^{8}^	0.86911^{11}^	0.86551^{9}^	0.86808^{10}^	0.84852^{6}^	0.85695^{7}^	0.77527^{2}^	0.83714^{5}^	0.90059^{12}^	0.2591^{1}^
		$\overset{ˆ}{\sigma }$	1.28595^{9}^	1.27324^{8}^	1.3114^{12}^	1.12284^{3}^	1.30668^{11}^	1.29634^{10}^	1.25176^{7}^	1.2066^{5}^	1.115^{2}^	1.165^{4}^	1.24279^{6}^	0.32009^{1}^
		$\overset{ˆ}{\omega }$	0.04705^{1}^	0.05027^{3}^	0.05685^{7}^	0.05168^{5}^	0.05743^{8}^	0.05438^{6}^	0.05056^{4}^	0.05871^{9}^	0.06635^{11}^	0.06272^{10}^	0.0804^{12}^	0.04994^{2}^
	MRE	$\overset{ˆ}{\eta }$	0.38044^{2}^	0.38672^{4}^	0.39234^{7}^	0.39475^{9}^	0.39565^{10}^	0.39298^{8}^	0.38967^{5}^	0.39003^{6}^	0.368^{1}^	0.38493^{3}^	0.40556^{11.5}^	0.40556^{11.5}^
		$\overset{ˆ}{\sigma }$	0.29364^{8}^	0.29134^{6}^	0.30008^{9}^	0.28075^{3}^	0.30049^{11}^	0.30011^{10}^	0.29264^{7}^	0.28413^{5}^	0.27723^{2}^	0.28262^{4}^	3.11807^{12}^	0.13742^{1}^
		$\overset{ˆ}{\omega }$	0.08684^{2}^	0.09005^{3}^	0.0956^{7}^	0.09236^{5}^	0.09589^{8}^	0.09351^{6}^	0.09013^{4}^	0.0972^{9}^	0.10321^{11}^	0.10023^{10}^	2.03201^{12}^	0.08599^{1}^
	∑*Ranks*		51^{2}^	56^{3}^	98^{9}^	71^{6.5}^	117^{11}^	100^{10}^	67^{5}^	82^{8}^	58^{4}^	71^{6.5}^	131^{12}^	34^{1}^

150	BIAS	$\overset{ˆ}{\eta }$	0.73171^{3}^	0.74452^{6}^	0.76365^{10}^	0.74306^{4}^	0.77184^{11}^	0.75924^{8}^	0.76103^{9}^	0.75363^{7}^	0.70601^{2}^	0.74408^{5}^	0.79562^{12}^	0.31625^{1}^
		$\overset{ˆ}{\sigma }$	0.8207^{6}^	0.8229^{7}^	0.85707^{10}^	0.76543^{3}^	0.87039^{11}^	0.87166^{12}^	0.82741^{8}^	0.80898^{5}^	0.76502^{2}^	0.78374^{4}^	0.83299^{9}^	0.34834^{1}^
		$\overset{ˆ}{\omega }$	0.1386^{2}^	0.13986^{4}^	0.15038^{7}^	0.14115^{5}^	0.1561^{10}^	0.13887^{3}^	0.14141^{6}^	0.15544^{9}^	0.15886^{11}^	0.1542^{8}^	0.18644^{12}^	0.12922^{1}^
	MSE	$\overset{ˆ}{\eta }$	0.75428^{3}^	0.78712^{4}^	0.82254^{10}^	0.79284^{5}^	0.8355^{11}^	0.82044^{9}^	0.81812^{8}^	0.80527^{7}^	0.72767^{2}^	0.79497^{6}^	0.87431^{12}^	0.19137^{1}^
		$\overset{ˆ}{\sigma }$	1.14784^{8}^	1.12805^{6}^	1.21877^{10}^	0.96311^{2}^	1.25171^{12}^	1.24939^{11}^	1.14322^{7}^	1.10836^{5}^	0.98021^{3}^	1.02575^{4}^	1.17269^{9}^	0.22527^{1}^
		$\overset{ˆ}{\omega }$	0.02981^{2}^	0.03031^{3}^	0.0352^{7}^	0.03063^{5}^	0.03752^{10}^	0.03037^{4}^	0.03094^{6}^	0.03715^{9}^	0.03944^{11}^	0.03674^{8}^	0.05367^{12}^	0.02759^{1}^
	MRE	$\overset{ˆ}{\eta }$	0.36586^{2}^	0.37226^{5}^	0.38183^{9}^	0.37153^{3}^	0.38592^{10}^	0.37962^{7}^	0.38051^{8}^	0.37682^{6}^	0.353^{1}^	0.37204^{4}^	0.39781^{11.5}^	0.39781^{11.5}^
		$\overset{ˆ}{\sigma }$	0.27357^{6}^	0.2743^{7}^	0.28569^{9}^	0.25514^{3}^	0.29013^{10}^	0.29055^{11}^	0.2758^{8}^	0.26966^{5}^	0.25501^{2}^	0.26125^{4}^	3.0744^{12}^	0.11611^{1}^
		$\overset{ˆ}{\omega }$	0.0693^{2}^	0.06993^{4}^	0.07519^{7}^	0.07057^{5}^	0.07805^{10}^	0.06944^{3}^	0.07071^{6}^	0.07772^{9}^	0.07943^{11}^	0.0771^{8}^	2.02091^{12}^	0.06461^{1}^
	∑*Ranks*		44^{2}^	62^{5}^	104^{10}^	46^{3}^	125^{11}^	89^{9}^	88^{8}^	82^{7}^	59^{4}^	67^{6}^	137^{12}^	33^{1}^
250	BIAS	$\overset{ˆ}{\eta }$	0.68231^{2}^	0.72173^{7}^	0.74035^{9}^	0.69316^{4}^	0.74799^{10}^	0.75253^{11}^	0.72122^{6}^	0.72187^{8}^	0.68759^{3}^	0.71565^{5}^	0.77972^{12}^	0.27055^{1}^
		$\overset{ˆ}{\sigma }$	0.74648^{5}^	0.77642^{8}^	0.82783^{10}^	0.69325^{2}^	0.82808^{11}^	0.85969^{12}^	0.76905^{7}^	0.75696^{6}^	0.72706^{4}^	0.72327^{3}^	0.77896^{9}^	0.30007^{1}^
		$\overset{ˆ}{\omega }$	0.11558^{3}^	0.11916^{6}^	0.12259^{7}^	0.11712^{4}^	0.12583^{8}^	0.11531^{2}^	0.11748^{5}^	0.12928^{10}^	0.13486^{11}^	0.12755^{9}^	0.166^{12}^	0.10072^{1}^
	MSE	$\overset{ˆ}{\eta }$	0.67646^{2}^	0.74737^{7}^	0.77785^{9}^	0.70478^{4}^	0.79355^{10}^	0.80216^{11}^	0.74319^{5}^	0.74997^{8}^	0.69893^{3}^	0.74487^{6}^	0.84738^{12}^	0.14347^{1}^
		$\overset{ˆ}{\sigma }$	0.97701^{5}^	1.0246^{8}^	1.12702^{10}^	0.80387^{2}^	1.14765^{11}^	1.21371^{12}^	0.99721^{7}^	0.98618^{6}^	0.91596^{4}^	0.87153^{3}^	1.03761^{9}^	0.16869^{1}^
		$\overset{ˆ}{\omega }$	0.02059^{3}^	0.02183^{6}^	0.02337^{7}^	0.0209^{4}^	0.02442^{8}^	0.02053^{2}^	0.02122^{5}^	0.0258^{10}^	0.02845^{11}^	0.02525^{9}^	0.04179^{12}^	0.0165^{1}^
	MRE	$\overset{ˆ}{\eta }$	0.34115^{1}^	0.36086^{6}^	0.37018^{8}^	0.34658^{3}^	0.374^{9}^	0.37626^{10}^	0.36061^{5}^	0.36094^{7}^	0.34379^{2}^	0.35782^{4}^	0.38986^{11.5}^	0.38986^{11.5}^
		$\overset{ˆ}{\sigma }$	0.24883^{5}^	0.25881^{8}^	0.27594^{9}^	0.23108^{2}^	0.27603^{10}^	0.28656^{11}^	0.25635^{7}^	0.25232^{6}^	0.24235^{4}^	0.24109^{3}^	3.055^{12}^	0.10002^{1}^
		$\overset{ˆ}{\omega }$	0.05779^{3}^	0.05958^{6}^	0.0613^{7}^	0.05856^{4}^	0.06292^{8}^	0.05766^{2}^	0.05874^{5}^	0.06464^{10}^	0.06743^{11}^	0.06377^{9}^	2.01237^{12}^	0.05036^{1}^
	∑*Ranks*		38^{2.5}^	82^{7}^	100^{10}^	38^{2.5}^	112^{11}^	96^{9}^	69^{5}^	94^{8}^	70^{6}^	67^{4}^	137^{12}^	33^{1}^

400	BIAS	$\overset{ˆ}{\eta }$	0.63198^{3}^	0.67151^{7}^	0.71879^{10}^	0.63151^{2}^	0.71469^{9}^	0.73149^{11}^	0.66859^{5}^	0.68303^{8}^	0.64976^{4}^	0.67007^{6}^	0.74003^{12}^	0.23561^{1}^
		$\overset{ˆ}{\sigma }$	0.66577^{5}^	0.70178^{7}^	0.78409^{10}^	0.6172^{2}^	0.78564^{11}^	0.8266^{12}^	0.71325^{8}^	0.69979^{6}^	0.65796^{4}^	0.65788^{3}^	0.72608^{9}^	0.25504^{1}^
		$\overset{ˆ}{\omega }$	0.09788^{2}^	0.1016^{6}^	0.10656^{7}^	0.09798^{3}^	0.10743^{8}^	0.09904^{4}^	0.10126^{5}^	0.11446^{11}^	0.11363^{10}^	0.11038^{9}^	0.14856^{12}^	0.08122^{1}^
	MSE	$\overset{ˆ}{\eta }$	0.59023^{2}^	0.67131^{7}^	0.7426^{10}^	0.60505^{3}^	0.73765^{9}^	0.76791^{11}^	0.65456^{5}^	0.6821^{8}^	0.643^{4}^	0.67079^{6}^	0.77144^{12}^	0.11037^{1}^
		$\overset{ˆ}{\sigma }$	0.78972^{5}^	0.85912^{7}^	1.03941^{10}^	0.65875^{2}^	1.05352^{11}^	1.13259^{12}^	0.87764^{8}^	0.84787^{6}^	0.76969^{4}^	0.74199^{3}^	0.91866^{9}^	0.12205^{1}^
		$\overset{ˆ}{\omega }$	0.01468^{2}^	0.01581^{5}^	0.01754^{7}^	0.01469^{3}^	0.01775^{8}^	0.01499^{4}^	0.01585^{6}^	0.01994^{11}^	0.01973^{10}^	0.01886^{9}^	0.03316^{12}^	0.01058^{1}^
	MRE	$\overset{ˆ}{\eta }$	0.31599^{2}^	0.33576^{6}^	0.35939^{9}^	0.31576^{1}^	0.35735^{8}^	0.36574^{10}^	0.33429^{4}^	0.34152^{7}^	0.32488^{3}^	0.33504^{5}^	0.37001^{11.5}^	0.37001^{11.5}^
		$\overset{ˆ}{\sigma }$	0.22192^{5}^	0.23393^{7}^	0.26136^{9}^	0.20573^{2}^	0.26188^{10}^	0.27553^{11}^	0.23775^{8}^	0.23326^{6}^	0.21932^{4}^	0.21929^{3}^	3.04036^{12}^	0.08501^{1}^
		$\overset{ˆ}{\omega }$	0.04894^{2}^	0.0508^{6}^	0.05328^{7}^	0.04899^{3}^	0.05372^{8}^	0.04952^{4}^	0.05063^{5}^	0.05723^{11}^	0.05681^{10}^	0.05519^{9}^	2.00592^{12}^	0.04061^{1}^
	∑*Ranks*		37^{3}^	77^{7}^	104^{9.5}^	27^{1}^	108^{11}^	104^{9.5}^	71^{6}^	98^{8}^	70^{4.5}^	70^{4.5}^	137^{12}^	33^{2}^

500	BIAS	$\overset{ˆ}{\eta }$	0.59951^{2}^	0.63736^{4}^	0.70411^{9}^	0.60696^{3}^	0.7123^{10}^	0.71262^{11}^	0.64217^{6}^	0.66404^{8}^	0.63928^{5}^	0.64549^{7}^	0.74712^{12}^	0.21688^{1}^
		$\overset{ˆ}{\sigma }$	0.63222^{4}^	0.67084^{6}^	0.77556^{11}^	0.57625^{2}^	0.76946^{10}^	0.80288^{12}^	0.67523^{7}^	0.68178^{8}^	0.64693^{5}^	0.62505^{3}^	0.72026^{9}^	0.23561^{1}^
		$\overset{ˆ}{\omega }$	0.09299^{4}^	0.09472^{6}^	0.09985^{7}^	0.09165^{3}^	0.10265^{9}^	0.09097^{2}^	0.0931^{5}^	0.10728^{11}^	0.10675^{10}^	0.09987^{8}^	0.14602^{12}^	0.07146^{1}^
	MSE	$\overset{ˆ}{\eta }$	0.54348^{2}^	0.60865^{4}^	0.71721^{9}^	0.56799^{3}^	0.72896^{10}^	0.72958^{11}^	0.61587^{5}^	0.65015^{8}^	0.62053^{6}^	0.62566^{7}^	0.79122^{12}^	0.09432^{1}^
		$\overset{ˆ}{\sigma }$	0.72404^{4}^	0.79083^{6}^	1.0264^{11}^	0.57782^{2}^	0.99881^{10}^	1.0688^{12}^	0.80133^{7}^	0.81127^{8}^	0.74836^{5}^	0.67086^{3}^	0.90304^{9}^	0.10941^{1}^
		$\overset{ˆ}{\omega }$	0.01316^{4}^	0.01384^{6}^	0.01529^{7}^	0.01283^{3}^	0.01605^{9}^	0.01273^{2}^	0.01334^{5}^	0.01739^{10}^	0.01756^{11}^	0.01555^{8}^	0.03178^{12}^	0.0083^{1}^
	MRE	$\overset{ˆ}{\eta }$	0.29976^{1}^	0.31868^{3}^	0.35205^{8}^	0.30348^{2}^	0.35615^{9}^	0.35631^{10}^	0.32109^{5}^	0.33202^{7}^	0.31964^{4}^	0.32275^{6}^	0.37356^{11.5}^	0.37356^{11.5}^
		$\overset{ˆ}{\sigma }$	0.21074^{4}^	0.22361^{6}^	0.25852^{10}^	0.19208^{2}^	0.25649^{9}^	0.26763^{11}^	0.22508^{7}^	0.22726^{8}^	0.21564^{5}^	0.20835^{3}^	3.04002^{12}^	0.07854^{1}^
		$\overset{ˆ}{\omega }$	0.0465^{4}^	0.04736^{6}^	0.04992^{7}^	0.04583^{3}^	0.05133^{9}^	0.04549^{2}^	0.04655^{5}^	0.05364^{11}^	0.05337^{10}^	0.04993^{8}^	2.00559^{12}^	0.03573^{1}^
	∑*Ranks*		38^{3}^	62^{4}^	104^{9}^	30^{1}^	112^{11}^	96^{8}^	69^{5}^	105^{10}^	80^{7}^	70^{6}^	137^{12}^	33^{2}^

**Table 6:** Partial and overall ranks of all the methods of estimating proposed distribution by various values of model parameters.

Parameter	*n*	*EM*_1_	*EM*_2_	*EM*_3_	*EM*_4_	*EM*_5_	*EM*_6_	*EM*_7_	*EM*_8_	*EM*_9_	*EM*_10_	*EM*_11_	*EM*_12_
*η* = 2.5, *σ* = 1.5, *ω* = 0.75	20	3.0	4.0	10.0	8.0	11.0	7.0	5.5	9.0	2.0	5.5	12.0	1.0
	80	2.0	4.0	11.0	3.0	10.0	8.0	7.0	9.0	5.0	6.0	12.0	1.0
	150	3.0	7.0	10.0	2.0	11.0	8.5	5.0	8.5	4.0	6.0	12.0	1.0
	250	3.0	7.0	9.0	1.0	11.0	10.0	5.0	8.0	6.0	4.0	12.0	2.0
	400	3.0	6.0	9.0	1.0	10.0	11.0	5.0	8.0	7.0	4.0	12.0	2.0
	500	3.0	6.0	8.0	1.0	11.0	10.0	5.0	9.0	7.0	4.0	12.0	2.0

*η* = 1.5, *σ* = 2.5, *ω* = 0.9	20	1.0	3.0	7.0	9.5	11.0	8.0	6.0	4.5	4.5	9.5	12.0	2.0
	80	2.0	6.0	11.0	3.0	10.0	7.0	5.0	8.5	4.0	8.5	12.0	1.0
	150	2.0	7.0	10.0	3.0	11.0	9.0	6.0	8.0	4.0	5.0	12.0	1.0
	250	2.0	4.0	11.0	3.0	10.0	8.0	6.0	9.0	7.0	5.0	12.0	1.0
	400	3.0	7.0	9.0	1.0	11.0	10.0	5.0	8.0	6.0	4.0	12.0	2.0
	500	2.0	5.0	11.0	1.0	10.0	9.0	6.0	8.0	7.0	4.0	12.0	3.0

*η* = 0.5, *σ* = 2.0, *ω* = 1.5	20	1.0	2.0	9.0	7.0	12.0	3.0	6.0	8.0	5.0	10.0	11.0	4.0
	80	2.0	7.0	11.0	1.0	10.0	9.0	6.0	8.0	4.0	5.0	12.0	3.0
	150	2.0	4.0	11.0	1.0	10.0	9.0	7.0	8.0	6.0	5.0	12.0	3.0
	250	3.0	4.5	11.0	1.0	10.0	9.0	7.0	8.0	6.0	4.5	12.0	2.0
	400	3.0	4.0	9.0	1.0	10.0	11.0	5.0	8.0	6.0	7.0	12.0	2.0
	500	3.0	4.0	10.0	1.0	9.0	11.0	5.0	7.0	6.0	8.0	12.0	2.0

*η* = 0.9, *σ* = 4.0, *ω* = 2.5	20	2.0	1.0	8.0	9.0	11.0	10.0	7.0	6.0	4.0	5.0	12.0	3.0
	80	1.0	6.0	10.0	2.0	9.0	11.0	3.0	8.0	4.0	7.0	12.0	5.0
	150	1.5	4.5	11.0	1.5	10.0	8.0	6.0	9.0	4.5	7.0	12.0	3.0
	250	3.0	6.0	10.0	2.0	11.0	9.0	7.0	8.0	4.0	5.0	12.0	1.0
	400	3.0	6.0	11.0	1.0	9.5	9.5	7.0	8.0	4.0	5.0	12.0	2.0
	500	3.0	7.0	10.0	1.0	11.0	8.0	4.0	9.0	5.0	6.0	12.0	2.0

*η* = 2.0, *σ* = 3.0, *ω* = 2.0	20	1.0	2.0	9.0	10.0	11.0	5.0	6.0	7.0	4.0	8.0	12.0	3.0
	80	2.0	3.0	9.0	6.5	11.0	10.0	5.0	8.0	4.0	6.5	12.0	1.0
	150	2.0	5.0	10.0	3.0	11.0	9.0	8.0	7.0	4.0	6.0	12.0	1.0
	250	2.5	7.0	10.0	2.5	11.0	9.0	5.0	8.0	6.0	4.0	12.0	1.0
	400	3.0	7.0	9.5	1.0	11.0	9.5	6.0	8.0	4.5	4.5	12.0	2.0
	500	3.0	4.0	9.0	1.0	11.0	8.0	5.0	10.0	7.0	6.0	12.0	2.0

∑ Ranks		70.0	150.0	293.5	89.0	315.5	263.5	171.5	240.5	151.5	175.0	359.0	61.0
Overall Rank		2	4	10	3	11	9	6	8	5	7	12	1

## 7 Real data analysis

In this section, we have employed two real datasets to demonstrate the superior flexibility of the MGExED compared to other established probability distributions in effectively modeling these real-world datasets. The analyzed first real data was studied by Cordeiro and Brito [9], and it pertains to the total milk production observed during the initial calving of 107 cows belonging to the SINDI breed. These cows are owned by the Carnaúba farm, a property under the ownership of Agropecuária Manoel Dantas Ltda (AMDA), situated in Taperoá city, Paraíba, Brazil. The second one reports the failure times of 20 components. It was introduced by Murthy et al. [26] and studied by Srivastava [33].

To illustrate the remarkable flexibility of the MGExED, we will undertake a comprehensive comparative analysis. This analysis will juxtapose the proposed model with widely recognized and established models. The selection of these well-known models for comparison is not arbitrary, but we choose them as they are extensions of our baseline model (exponential distribution). These diverse sets of models serve as a comprehensive benchmark for evaluating the proposed model's capabilities. Through this comparative exploration, we aspire to provide a comprehensive and well-informed analysis that underscores the unique features and advantages of the proposed model in the realm of practical modeling. The proposed model is compared with the following existing models: exponentiated exponential distribution (EXED), exponential distribution (ED), Weibull distribution (WD), alpha power exponential distribution (APED) [21], alpha power exponentiated exponential distribution (APEXED) [1], beta exponential distribution (BED) [27], gamma exponentiated exponential distribution (GEXED) [29], generalized log-logistic exponential distribution (GLLED) [2], generalized odd log-logistic exponential distribution (GOLLED) [2], Marshall Olkin alpha power exponential distribution (MOAPED) [28], and Marshall Olkin logistic exponential distribution (MOLED) [22].

In determining the most suitable model for the two real datasets, we employ a set of rigorous analytical criteria. These criteria encompass a range of statistical measures, including the Akaike information criterion ($Z_{1}$), the corrected Akaike information criterion ($Z_{2}$), the Bayesian information criterion ($Z_{3}$), and the Hannan information criterion ($Z_{4}$). Additionally, our choice is underpinned by a thorough assessment of the model's overall goodness-of-fit, such as the Anderson-Darling statistic ($Z_{5}$), the Cramér-von Mises statistic ($Z_{6}$), and the Kolmogorov-Smirnov statistic ($Z_{7}$) alongside its accompanying p-value ($Z_{7}(p)$). The combined evaluation of these measures enables us to make a well-informed decision regarding the most appropriate model for fitting these datasets.

**Figure 2:**
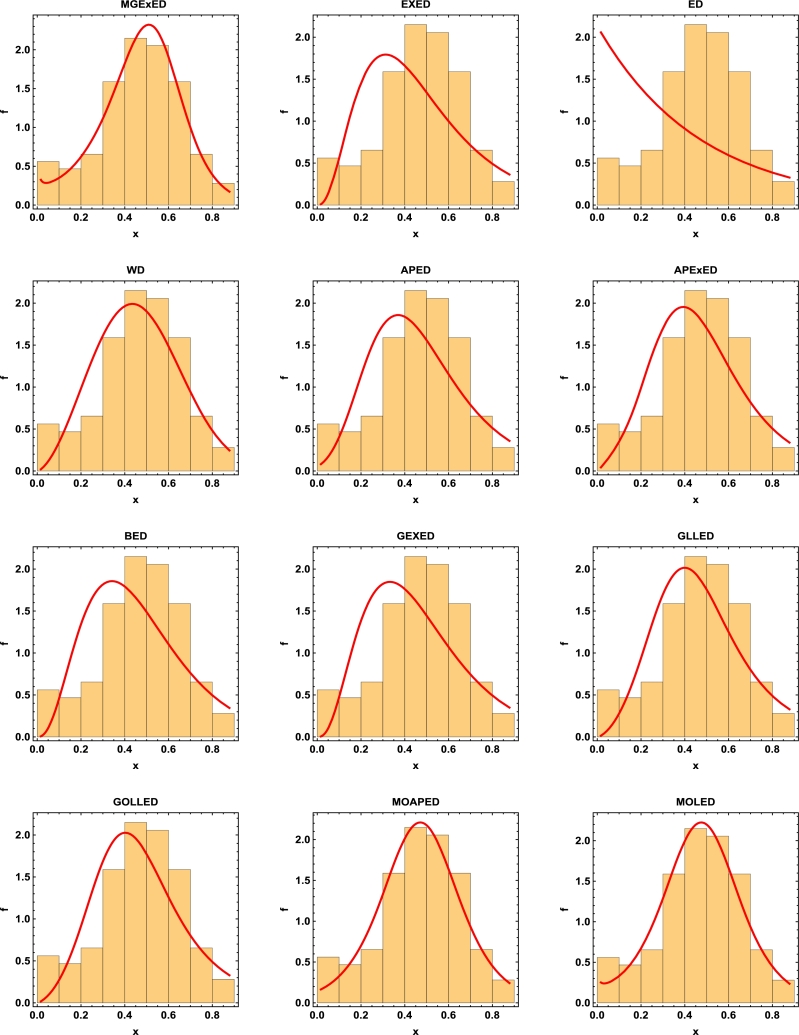
Histogram of the milk real dataset with the fitted PDFs of all compared models.

**Figure 3:**
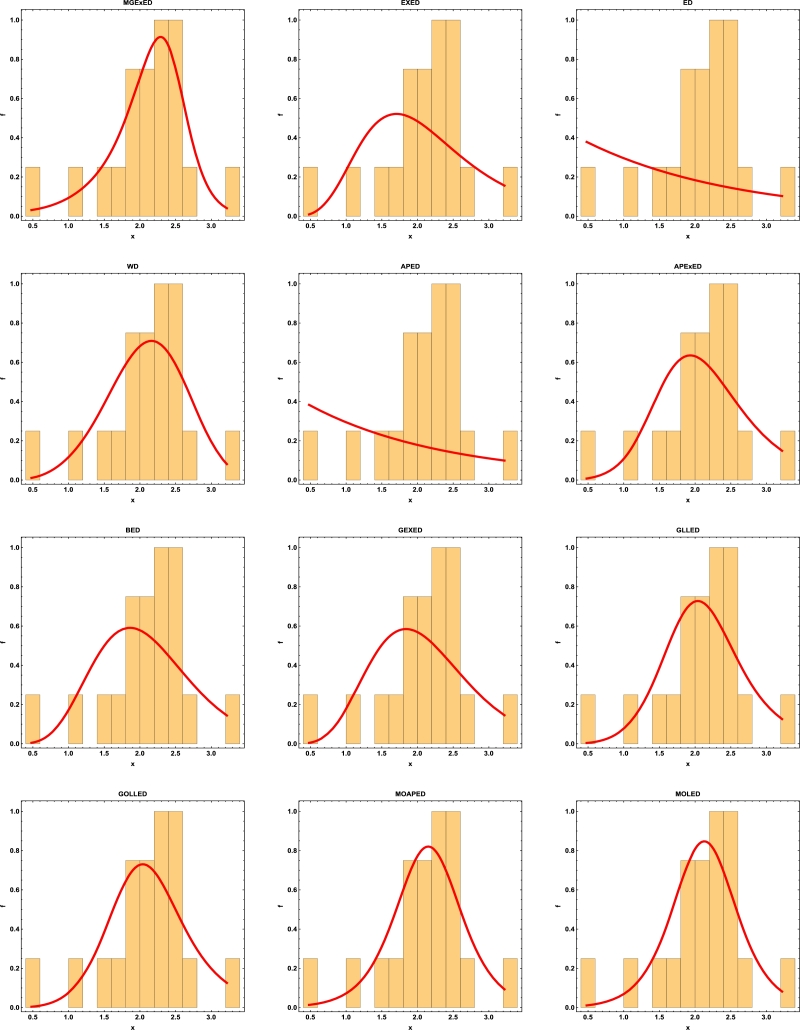
Histogram of the failure times real dataset with the fitted PDFs of all compared models.

**Figure 4:**
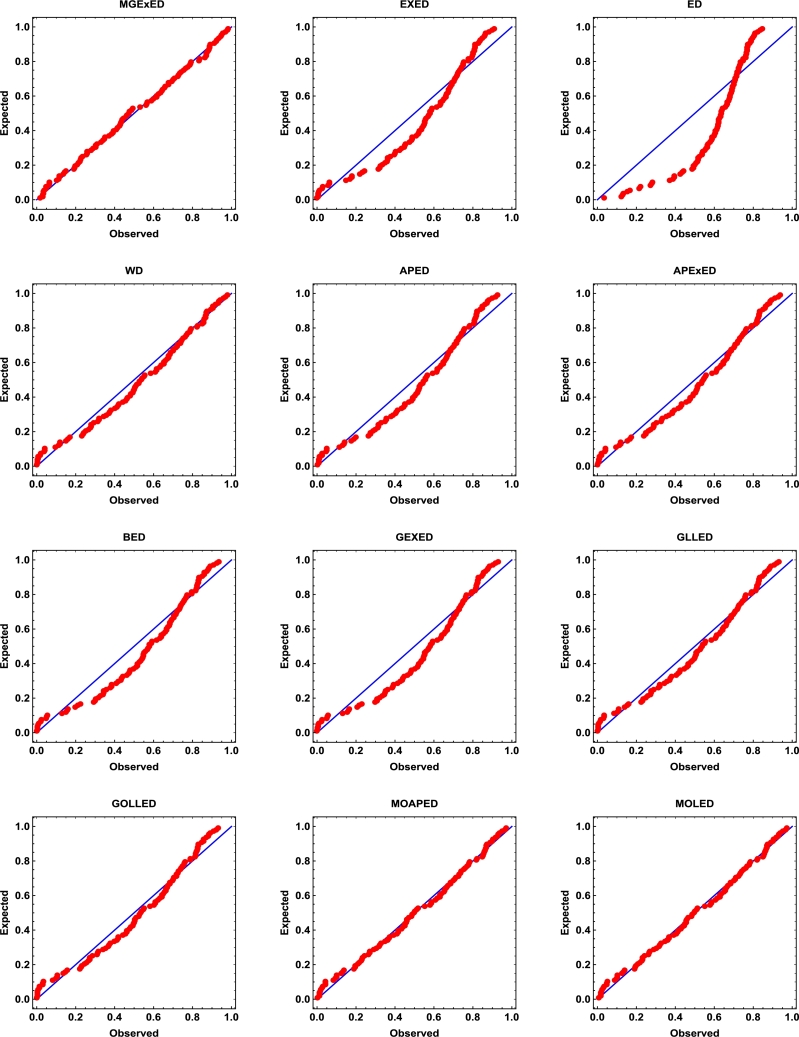
P-P plots of all compared models for the milk real dataset.

**Figure 5:**
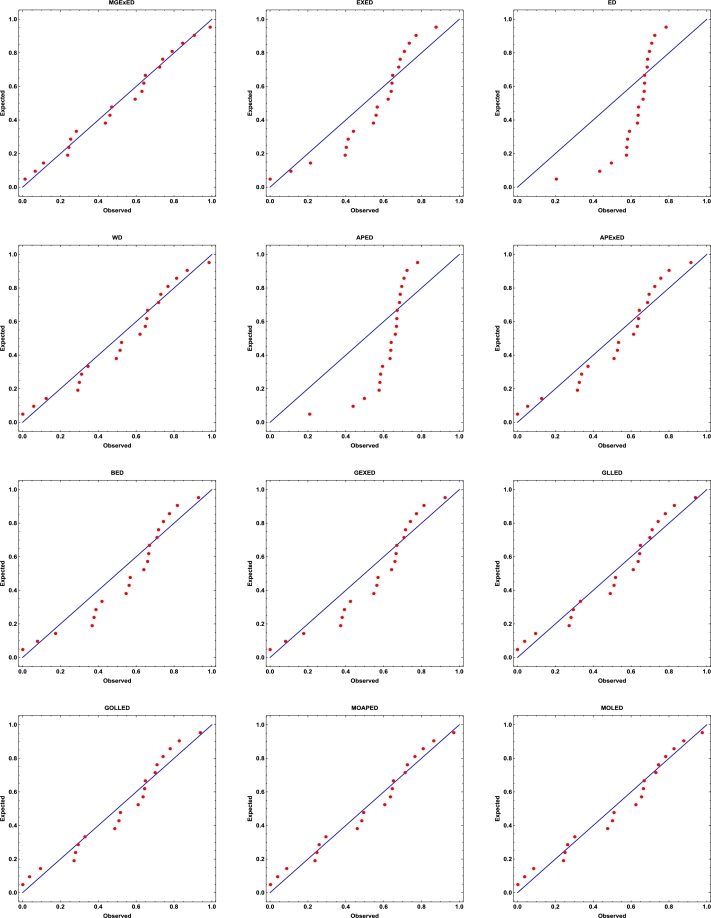
P-P plots of all compared models for the failure times real dataset.

**Figure 6:**
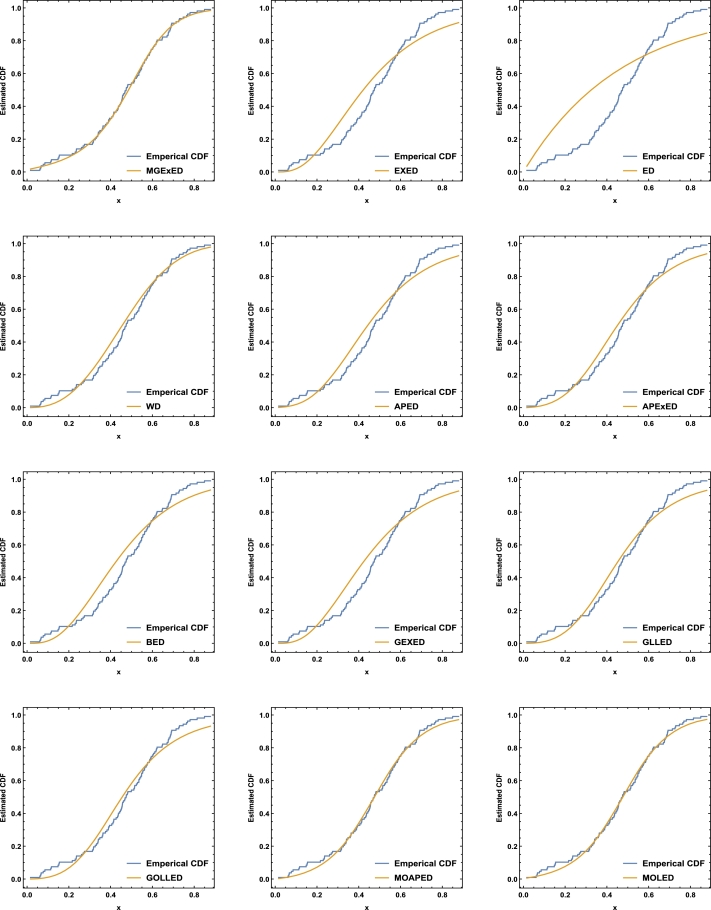
Fitted CDFs of all compared models for the milk real dataset.

**Figure 7:**
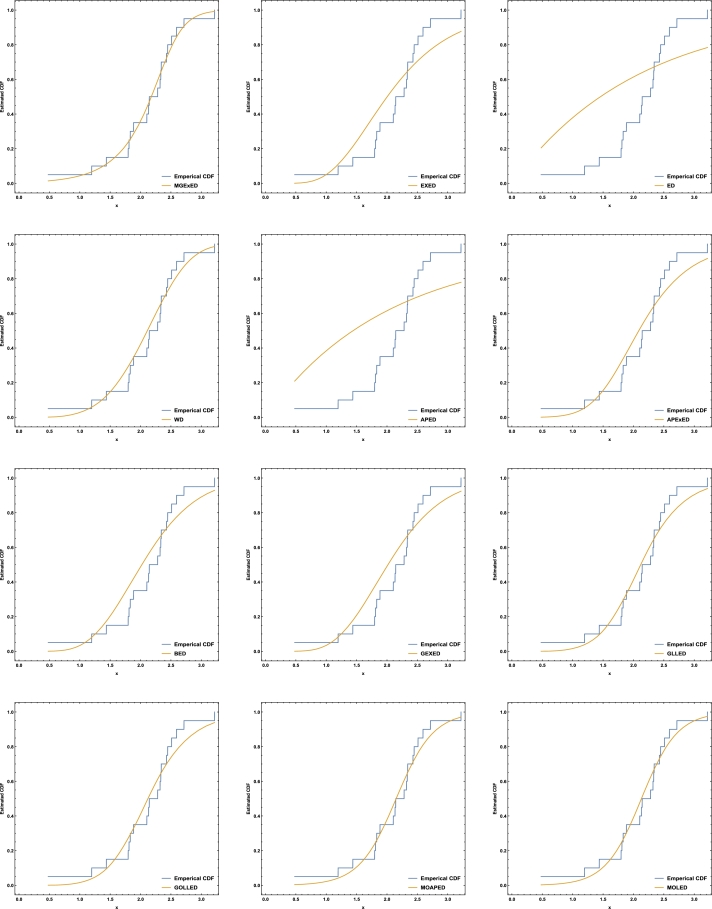
Fitted CDFs of all compared models for the failure times real dataset.

**Figure 8:**
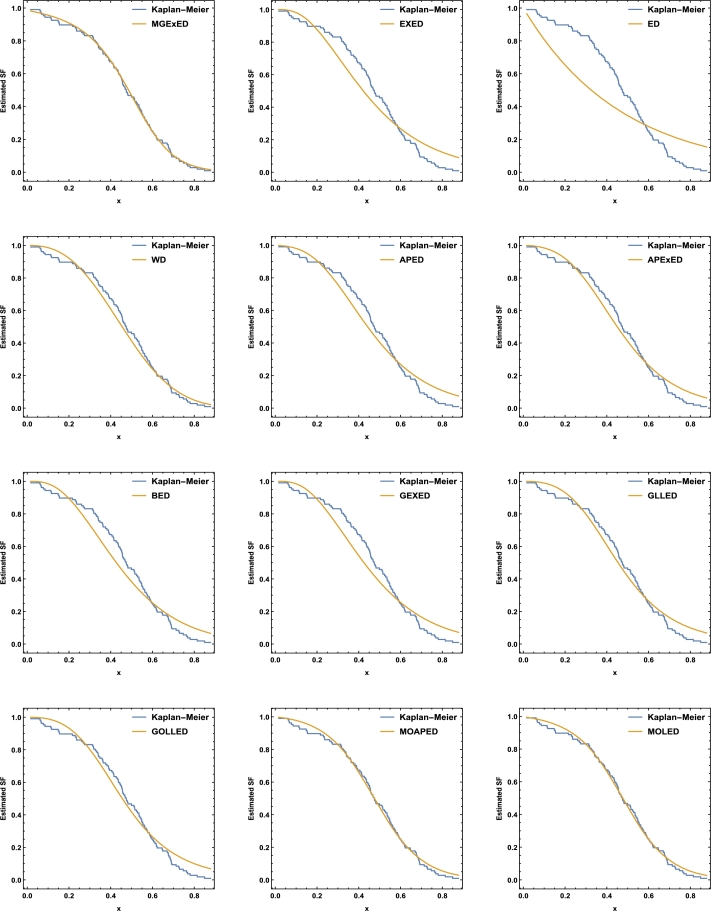
Fitted SFs of all compared models for the milk real dataset.

**Figure 9:**
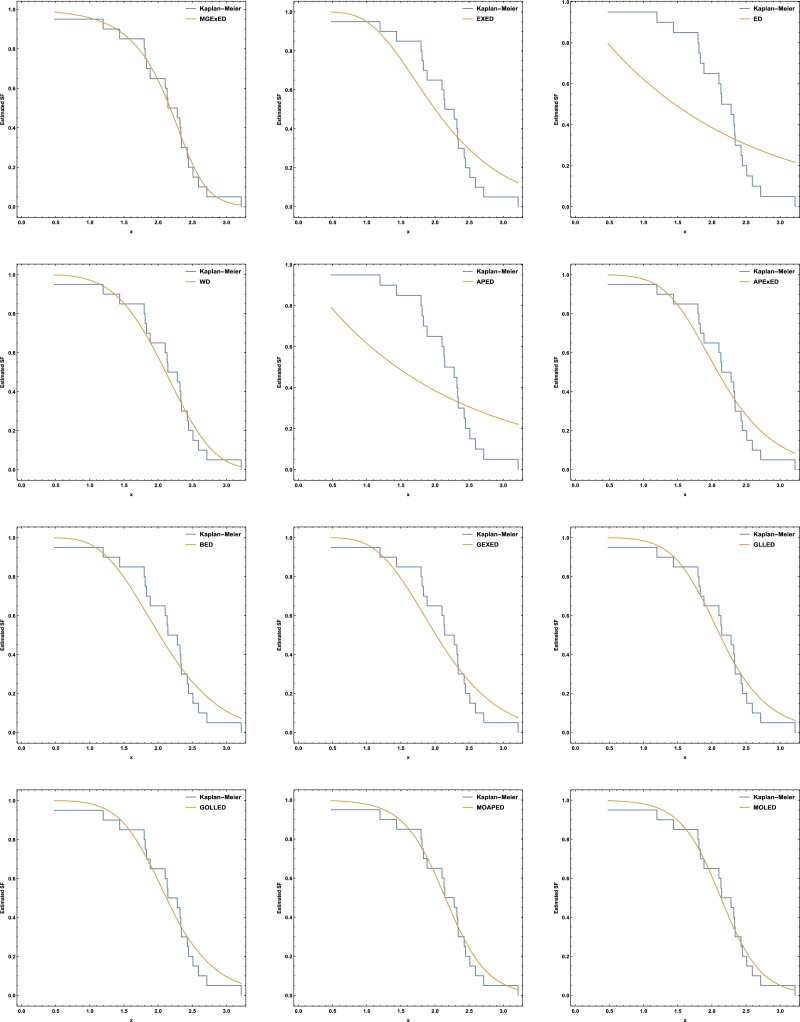
Fitted SFs of all compared models for the failure times real dataset.

**Figure 10:**
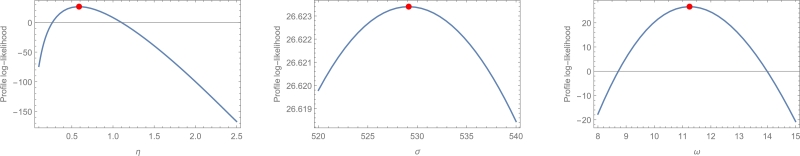
The profile of the log-likelihood functions for the MGExED estimators of the milk real dataset.

**Figure 11:**
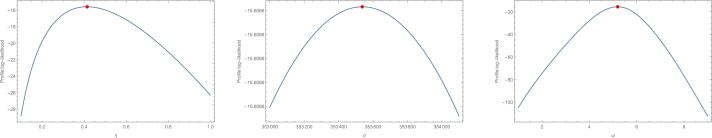
The profile of the log-likelihood functions for the MGExED estimators of the failure times real dataset.

Comprehensive analytical measurements and estimators of all compared models with corresponding standard errors (SE) have been provided for the two datasets under consideration, as detailed in Table 7, Table 8. The evaluation of the two datasets allows us to infer that the MGExED model outperforms its equivalent counterparts. The effectiveness of the MGExED model is presented for the two datasets by the Probability-Probability (P-P) and the associated fitted PDFs, CDFs, and SFs plots, as illustrated in Figure 2, Figure 3, Figure 4, Figure 5, Figure 6, Figure 7, Figure 8, Figure 9, respectively. Figure 10, Figure 11 depict the log-likelihood function's behavior concerning the estimated parameters for the two datasets, revealing its unimodal nature.

**Table 7:** Analyzing the milk real dataset.

Model	−L	*Z*_1_	*Z*_2_	*Z*_3_	*Z*_4_	*Z*_5_	*Z*_6_	*Z*_7_	*Z*_7_(*p*)	Est. parameters (SEs)
MGExED	-26.6234	-47.2468	-47.0138	-39.2283	-43.9962	0.204399	0.0215862	0.0423402	0.990787	$\overset{ˆ}{\eta }=0.587026\;(0.182288)$
										$\overset{ˆ}{\sigma }=529.113\;(801.313)$
										$\overset{ˆ}{\omega }=11.2442\;(1.93482)$

EXED	-5.03875	-6.0775	-5.96212	-0.731843	-3.91044	4.6866	0.812509	0.14765	0.0188327	$\overset{ˆ}{b}=3.71391\;(0.565791)$
										$\overset{ˆ}{a}=4.2007\;(0.372788)$

ED	25.9508	53.9015	53.9396	56.5744	54.9851	16.1817	3.28606	0.319269	<0.00001	$\overset{ˆ}{a}=2.13287\;(0.206192)$

WD	-21.3475	-38.695	-38.5796	-33.3494	-36.528	1.48405	0.18946	0.083241	0.448686	$\overset{ˆ}{a}=2.60119\;(0.209828)$
										$\overset{ˆ}{b}=0.523604\;(0.0202481)$

APED	-13.5333	-23.0665	-22.9511	-17.7209	-20.8995	2.78821	0.424997	0.110444	0.146963	$\overset{ˆ}{\alpha }=620.449\;(580.44)$
										$\overset{ˆ}{a}=5.04352\;(0.364154)$

APEXED	-15.9383	-25.8767	-25.6437	-17.8582	-22.6261	2.17382	0.283738	0.0945461	0.294342	$\overset{ˆ}{\alpha }=51.7227\;(49.2804)$
										$\overset{ˆ}{a}=5.64526\;(0.465145)$
										$\overset{ˆ}{c}=2.27947\;(0.659045)$

BED	-9.42041	-12.8408	-12.6078	-4.82234	-9.59023	3.81139	0.642033	0.136598	0.0368866	$\overset{ˆ}{\lambda }=0.122197\;(0.00145057)$
										$\overset{ˆ}{a}=3.68848\;(2.13081)$
										$\overset{ˆ}{b}=63.0646\;(0.482987)$

GEXED	-7.63627	-9.27253	-9.03952	-1.25404	-6.02194	4.11702	0.698036	0.140492	0.0292824	$\overset{ˆ}{\lambda }=1.75232\;(0.863589)$
										$\overset{ˆ}{\alpha }=4.5486\;(0.828209)$
										$\overset{ˆ}{\delta }=3.25846\;(1.88927)$

GLLED	-13.287	-22.5739	-22.4586	-17.2283	-20.4069	2.32479	0.245252	0.0859314	0.408264	$\overset{ˆ}{\alpha }=2.52837\;(0.217084)$
										$\overset{ˆ}{\lambda }=1.52761\;(0.0712574)$

GOLLED	-13.5403	-21.0806	-20.8476	-13.0621	-17.83	2.26894	0.229458	0.0822204	0.464568	$\overset{ˆ}{\alpha }=3.18307\;(1.12357)$
										$\overset{ˆ}{\lambda }=1.04056\;(0.6398)$
										$\overset{ˆ}{\theta }=0.711445\;(0.347648)$

MOAPED	-25.4163	-44.8325	-44.5995	-36.814	-41.5819	0.602458	0.0446063	0.0583598	0.859375	$\overset{ˆ}{\alpha }=1.00004\;(1.16368)$
										$\overset{ˆ}{\lambda }=8.69506\;(0.787173)$
										$\overset{ˆ}{\theta }=60.9592\;(43.953)$

MOLED	-25.9499	-45.8997	-45.6667	-37.8812	-42.6491	0.453096	0.0367946	0.0529763	0.92482	$\overset{ˆ}{\alpha }=0.677303\;(0.293759)$
										$\overset{ˆ}{\lambda }=13.1136\;(5.97072)$
										$\overset{ˆ}{\theta }=68.4141\;(28.0376)$

**Table 8:** Analyzing the failure times real dataset.

Model	−L	*Z*_1_	*Z*_2_	*Z*_3_	*Z*_4_	*Z*_5_	*Z*_6_	*Z*_7_	*Z*_7_(*p*)	Est. parameters (SEs)
MGExED	15.6066	37.2132	38.7132	40.2004	37.7963	0.197059	0.0289998	0.093832	0.994586	$\overset{ˆ}{\eta }=0.413601\;(0.241525)$
										$\overset{ˆ}{\sigma }=353538\;(1.96004\times 10^{6})$
										$\overset{ˆ}{\omega }=5.19249\;(1.98973)$

EXED	22.6814	49.3628	50.0687	51.3543	49.7516	1.58043	0.274808	0.246169	0.177015	$\overset{ˆ}{b}=9.92397\;(4.1352)$
										$\overset{ˆ}{a}=1.3457\;(0.232923)$

ED	34.844	71.688	71.9102	72.6837	71.8823	5.03175	1.05077	0.424926	0.001459	$\overset{ˆ}{a}=0.476067\;(0.106452)$

WD	17.2824	38.5648	39.2707	40.5563	38.9536	0.52541	0.0788565	0.14448	0.797972	$\overset{ˆ}{a}=4.3044\;(0.774037)$
										$\overset{ˆ}{b}=2.29951\;(0.124371)$

APED	35.3799	74.7599	75.4658	76.7514	75.1487	5.1096	1.0679	0.427027	0.00135908	$\overset{ˆ}{\alpha }=1.6653\times 10^{-8}\;(8.05323\times 10^{-7})$
										$\overset{ˆ}{a}=0.0273975\;(0.0759763)$

APEXED	19.6534	45.3069	46.8069	48.2941	45.89	0.904035	0.136993	0.167298	0.63023	$\overset{ˆ}{\alpha }=135.849\;(287.505)$
										$\overset{ˆ}{a}=1.88449\;(0.306237)$
										$\overset{ˆ}{c}=7.61417\;(4.76305)$

BED	20.7296	47.4592	48.9592	50.4464	48.0423	1.19256	0.20309	0.218358	0.296014	$\overset{ˆ}{\lambda }=0.0079232\;(0.0000768)$
										$\overset{ˆ}{a}=8.76789\;(21.623)$
										$\overset{ˆ}{b}=522.942\;(2.7218)$

GEXED	21.051	48.102	49.602	51.0892	48.6851	1.24914	0.212899	0.223133	0.272279	$\overset{ˆ}{\lambda }=0.342938\;(0.42433)$
										$\overset{ˆ}{\alpha }=11.7346\;(5.06519)$
										$\overset{ˆ}{\delta }=8.4862\;(12.6959)$

GLLED	18.8472	41.6944	42.4003	43.6859	42.0832	0.682404	0.0861981	0.139428	0.831664	$\overset{ˆ}{\alpha }=4.38985\;(0.86477)$
										$\overset{ˆ}{\lambda }=0.327159\;(0.0200702)$

GOLLED	18.8107	43.6215	45.1215	46.6087	44.2046	0.676595	0.0849425	0.137975	0.840944	$\overset{ˆ}{\alpha }=5.49878\;(4.99304)$
										$\overset{ˆ}{\lambda }=0.22332\;(0.359352)$
										$\overset{ˆ}{\theta }=0.710997\;(0.915444)$

MOAPED	16.5683	39.1366	40.6366	42.1238	39.7197	0.370422	0.0498325	0.111599	0.964528	$\overset{ˆ}{\alpha }=1.0003\;(8.28536)$
										$\overset{ˆ}{\lambda }=3.27994\;(0.636574)$
										$\overset{ˆ}{\theta }=1157.19\;(5076.49)$

MOLED	16.7151	39.4302	40.9302	42.4174	40.0134	0.405582	0.0564253	0.126679	0.905267	$\overset{ˆ}{\alpha }=1.4286\;(0.504141)$
										$\overset{ˆ}{\lambda }=2.35712\;(0.849572)$
										$\overset{ˆ}{\theta }=1297.61\;(1336.3)$

## 8 Conclusion

In summary, this study has introduced a novel two-parameter generalized family of distributions by applying the mixture definition to the exponentiated G-family of generalized distributions. This newly proposed family builds upon the exponential distribution as a reference model and has been successfully extended to a three-parameter distribution. We thoroughly investigate various statistical properties of this extended distribution, including its quantile functions, moments, and order statistics. We employ several estimation methods to determine the model parameters and rigorously evaluate their performance through simulations with randomly generated datasets. These simulations provide valuable insights into the accuracy and reliability of the estimators. Additionally, the practical applicability and flexibility of the new model are demonstrated through its analysis of a real dataset, revealing its superior ability to fit diverse data compared to existing models in the literature. This comprehensive evaluation underscores the model's robustness and potential for broad application in statistical analysis.

## CRediT authorship contribution statement

**Alaa R. El-Alosey:** Writing – review & editing, Supervision, Project administration, Methodology. **Mohammed S. Alotaibi:** Writing – original draft, Software, Methodology. **Ahmed M. Gemeay:** Writing – review & editing, Writing – original draft, Software, Resources, Data curation.

## Declaration of Competing Interest

The authors declare that they have no known competing financial interests or personal relationships that could have appeared to influence the work reported in this paper.

## Data Availability

The original contributions presented in the study are included in the article/supplementary material, further inquiries can be directed to the corresponding author/s.
